# Unveiling the Unique Mitogenome Structure of *Phylloporus*: Implications for Phylogeny and Evolution in Boletaceae

**DOI:** 10.3390/jof11120831

**Published:** 2025-11-25

**Authors:** Jie-Yu Huang, Zhen Zhang, Ming-Wei Mao, Kuan Zhao, Shan Yang

**Affiliations:** Key Laboratory of Natural Microbial Medicine Research of Jiangxi Province, College of Life Science, Jiangxi Science and Technology Normal University, Nanchang 330013, China

**Keywords:** Basidiomycota, boletes, phylogenetic analysis, comparative genomics

## Abstract

The genus *Phylloporus* (Boletaceae, Boletales) is a group of ectomycorrhizal fungi, distinguished from other members of Boletaceae by its unique lamellate hymenophore. Although some molecular data exist for this genus, its mitogenomic characteristics remain poorly understood. In our study, we sequenced, assembled, and annotated the complete mitogenomes of eight species representing seven major subclades of Boletaceae collected in Jiangxi Province, China, with a focus on four *Phylloporus* species. We found that *Phylloporus* mitogenomes are circular, ranging in size from 35,117 bp to 38,908 bp, and contain 14–15 core protein-coding genes (PCGs), 24–28 tRNA genes, and 2 rRNA genes. Our comparative analysis revealed that *Phylloporus* species share many features, such as gene content, gene length, tRNA repertoire, and gene order, while Boletaceae as a whole shows a lot of diversity. Codon usage patterns are quite similar across the family. The Ka/Ks ratios of most 15 core PCGs were less than 1, suggesting these genes have been preserved through purifying selection over time. By using Bayesian inference (BI) and maximum likelihood (ML) methods and combining 28 other mitotic genomes in the NCBI database, our phylogenetic analysis produced highly consistent and well-supported trees (BPP ≥ 0.98, BS ≥ 71). It is noted that this family is divided into seven subfamilies, which is consistent with previous taxonomic studies. Altogether, our findings shed light on the unique features of *Phylloporus* and its connections to other members of Boletaceae. These findings not only provide valuable insights into the taxonomy, phylogeny, genetic diversity, and resource conservation of Boletaceae but also serve as a valuable genomic resource for future research.

## 1. Introduction

The genus *Phylloporus* was established by Quélet in 1888 [1], with *Ph. pelletieri* (Lév.) Quél. as type species. This genus is distinguished from other members of the family Boletaceae by its lamellate hymenophore. *Phylloporus* species are common edible and medicinal fungi that form ectomycorrhizal associations with both coniferous and broad-leaved trees [2]. This symbiotic relationship plays a crucial role in maintaining the stability of global forest ecosystems and the carbon cycle [3,4].

*Phylloporus* is widely distributed in tropical, subtropical, and temperate regions of the Northern Hemisphere. Jiangxi Province, with a subtropical humid climate in southern China [5], is suitable for the growth and reproduction of macrofungi. A total of 84 species belonging to 27 genera of Boletaceae have been reported here, including six lamellate boletes [6,7]. In recent years, we collected four *Phylloporus* species from Jiangxi Province.

The mitochondrial genome is becoming an important tool for studying species origin, evolution, and classification due to its high copy number, low mutation rate, and rapid evolutionary rate [8]. For example, the structural characteristics, gene sequences, and genetic information from protein-coding genes of mitochondrial genomes can serve as a potential alternative tool for fungal taxonomy and phylogenetic analysis [9,10]. Therefore, determining the mitogenome sequence and analyzing its basic features is of pivotal importance [11,12]. Indeed, although there are about forty thousand species of basidiomycetes, fewer than 120 fungal mitogenomes have been deposited in public databases such as GenBank, the Joint Genome Institute, and the European Nucleotide Archive [13]. Consequently, the mitochondrial characteristics of *Phylloporus* species remain unclear.

In this study, five specimens of *Phylloporus* collected from Jiangxi Province, China, were subjected to second-generation sequencing, assembly, and annotation. By analyzing the structural features of their mitogenomes, protein-coding genes, codon usage bias, and other characteristics, we aim to reveal their genetic traits and evolutionary adaptations. Furthermore, five specimens from Boletaceae (except for *Phylloporus*) were also newly sequenced. Combining those with 26 Boletaceae species available in GenBank and using Paxillaceae as an outgroup, a phylogenetic framework based on 28 mitogenomes was constructed to confirm the phylogenetic position of the genus *Phylloporus*.

## 2. Materials and Methods

### 2.1. Sample Collection and DNA Extraction

Nine specimens belonging to eight species of Boletaceae were collected from Jiangxi Province, China. They were first identified by their macroscopic characteristics, such as the pileus, stipe, and hymenophore, and their microscopic characteristics, such as the size of the spores. Additionally, molecular analyses further confirm their identity. The total DNA was extracted from basidiocarp tissues using the cetyltrimethylammonium bromide (CTAB) method [13]. The specimens are stored at the Cryptogamic Herbarium, Kunming Institute of Botany, Chinese Academy of Sciences (HKAS). The species names are as follows: *Phylloporus brunneiceps*, *Phylloporus grossus*, *Phylloporus rubrosquamosus*, *Phylloporus luxiensis*, *Austroboletus fusisporus*, *Chalciporus radiatus*, *Chiua viridula*, *Zangia olivaceobrunnea*; their corresponding specimen collection numbers are HKAS1059–HKAS1064 and HKAS105268–HKAS105270. They were successfully sequenced. Sequencing was performed by Sangon Biotech Co., Ltd. (Shanghai, China) to generate high-quality sequence reads.

### 2.2. Mitogenomes Assembly and Annotation

GetOrganelle v.1.6.2 software [14] was used for genome assembly with the fungal database (-F fungus_mt) to identify, filter, and assemble target-related reads. Annotation was conducted using the MITOS web server with the genetic code table 4 [15]. Annotated protein-coding genes (PCGs) were further optimized using the NCBI Open Reading Frame (ORF) Finder (https://www.ncbi.nlm.nih.gov/orffinder/, accessed on 5 December 2024). tRNA genes were identified using tRNAscan-SE v1.3.1 [16]. Intron types (if found) were verified with RNAweasel v5.2.1 [17]. Gene maps were generated using OGDRAW v1.3.1 [18]. tRNA genes were analyzed using the software tRNAscan-SE v.2.0.9 [19], and the secondary structures of tRNA genes were drawn with the software VARNA v.3-93 [20].

### 2.3. Sequence Analysis

The GC content of mitogenomes was calculated using Geneious v9.0.2. Synteny analysis was performed with the built-in Mauve plugin [21]. Start and stop codons of PCGs were identified using PhyloSuite v1.2.3 [22]. Base composition of the nine Boletaceae mitogenomes was analyzed with DNASTAR Lasergene v7.1 (http://www.dnastar.com/, accessed on 20 December 2024). Strand asymmetry was calculated as follows: AT skew = (A − T)/(A + T) and GC skew = (G − C)/(G + C) [23]. BLASTN searches were conducted to detect interspersed repeats or large intragenomic duplications (https://blast.ncbi.nlm.nih.gov/Blast.cgi/, accessed on 20 December 2024). Tandem repeats were identified using Tandem Repeats Finder [24]. Furthermore, TBtools-II and DnaSP v6 [25] were used to estimate the nonsynonymous (Ka) and synonymous (Ks) substitution rates of 15 core PCGs (atp6, atp8, atp9, cob, cox1, cox2, cox3, nad1, nad2, nad3, nad4, nad4L, nad5, nad6, and rps3) across the nine mitogenomes.

### 2.4. Phylogenetic Analysis

Mitochondrial genomes of nine specimens from Boletaceae, including five *Phylloporus* specimens, were newly sequenced for this study. Additionally, 19 publicly available mitogenomes were retrieved from NCBI based on previous studies [26,27,28,29,30], comprising 17 samples from Boletaceae and two from Paxillaceae (order Boletales) designated as the outgroup (Table 1). Phylogenetic analysis was performed using PhyloSuite v1.2.3 [22,31]. Fifteen core protein-coding genes (PCGs) were extracted and individually aligned with MAFFT as implemented in PhyloSuite [32]. The resulting alignments were trimmed, respectively, by the Gblock tool and then integrated into a single matrix using the sequence concatenation tool in PhyloSuite. The final concatenated alignment had a total length of 14,131 bp, containing 2016 parsimony-informative sites. Through the PartitionFinder2 software, by using the greedy search algorithm and the associated branch length and based on the Bayesian Information Criterion (BIC) and the modified Akaike Information Criterion (AICc), the GTR + I + G model was determined as the single partition and the best nucleotide replacement model. Phylogenetic trees were reconstructed using both Bayesian Inference (BI) and Maximum Likelihood (ML) methods. The BI analysis was conducted with two independent runs of four Markov chain Monte Carlo chains each for 1,000,000 generations, sampling every 1000 generations. The average standard deviation of split frequencies reached 0.005 (<0.01), indicating satisfactory convergence. Posterior probabilities (PP) were calculated for each node after discarding 25% of the samples as burn-in. The ML tree was inferred with IQ-TREE [33] within PhyloSuite, employing ultrafast bootstrap approximation with 10,000 replicates under an edge-linked partition model [34,35] to assess branch support. Final trees were visualized in FigTree v1.4.2 and aesthetically adjusted using Adobe Illustrator CS5.

### 2.5. Data Availability

The complete mitogenomes of the nine Boletaceae specimens were deposited in the GenBank database under the accession numbers of PV424033-PV424039, PQ821895, and PQ846490.

## 3. Results

### 3.1. Characterization of Mitogenomes in Phylloporus

**Figure 1:**
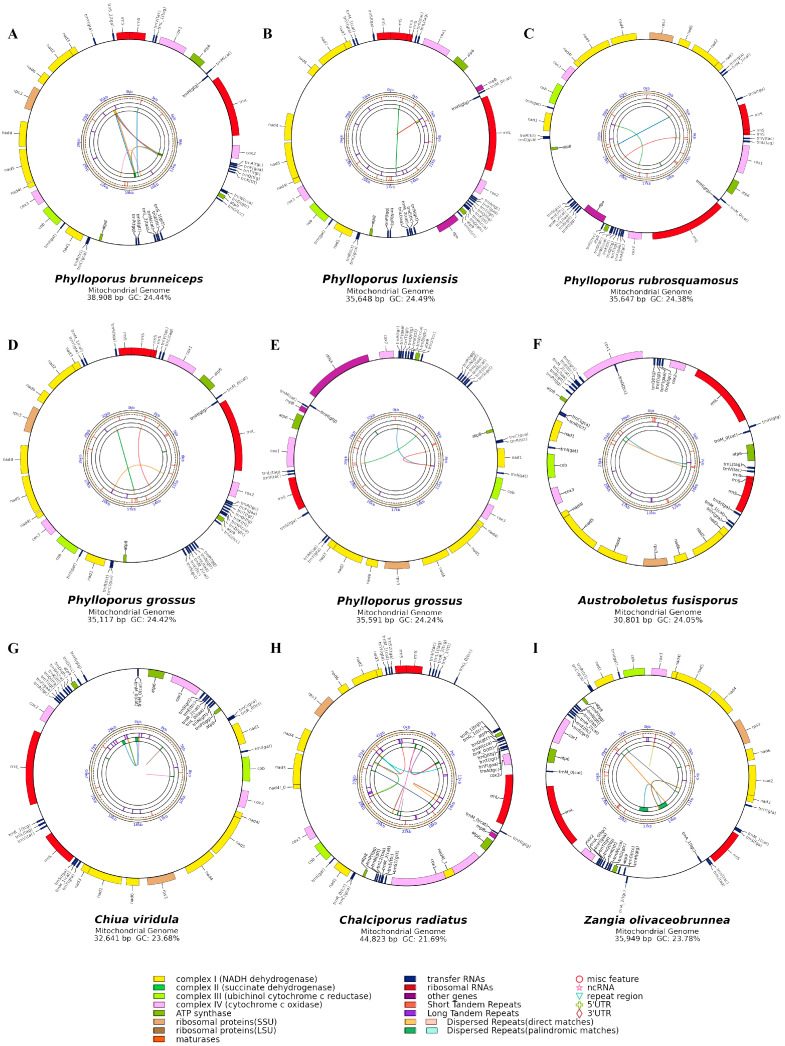
Circular maps of the nine mitogenomes from Boletaceae. (**A**) *Phylloporus brunneiceps* * HKAS105261; (**B**) *Phylloporus luxiensis* HKAS105263; (**C**) *Phylloporus rubrosquamosus* HKAS105259; (**D**) *Phylloporus grossus* HKAS105262; (**E**) *Phylloporus grossus* HKAS105260; (**F**) *Austroboletus fusisporus* HKAS105269; (**G**) *Chiua viridula* HKAS105270; (**H**) *Chalciporus radiatus* HKAS105264; (**I**) *Zangia olivaceobrunnea* HKAS105268). Each gene is represented by a corresponding colored block. Colored blocks outside the ring indicate genes located on the direct strand, while those inside the ring represent genes on the reverse strand. Colored lines for intraspecies collinearity analysis.

The complete mitogenomes of *Phylloporus* species were all circular DNA molecules (Figure 1A–F), with sizes ranging from 35,117 bp to 38,908 bp and an average size of 36,182 bp. These mitogenomes exhibited low GC content, ranging from 21.69% to 24.49%, with an average of 23.91%. All *Phylloporus* species showed relatively high GC content, each exceeding 24.24%. The AT skew values were positive across the genus, ranging from −0.03 to 0.06 and averaging 0.027. Except *Austroboletus fusisporus*, which showed a GC skew of 0.01, all other genomes displayed negative GC skew, ranging from −0.04 to −0.02(Table 2).

The mitogenomes contained 15 protein-coding genes (PCGs), including one ribosomal protein S3 (rps3), three ATP synthase subunits (atp6, atp8, and atp9), three cytochrome c oxidase subunits (cox1, cox2, and cox3), seven NADH dehydrogenase subunits (nad1, nad2, nad3, nad4, nad4L, nad5, and nad6), one cytochrome b (cob), and two ribosomal RNAs. The number of tRNA genes among the nine mitogenomes ranged from 20 to 26, with an average of 23. These tRNAs encode all 20 standard amino acids.

### 3.2. Analysis of Repetitive Sequences

Repetitive sequences can be classified into three main categories: simple sequence repeats (SSRs), tandem repeats, and dispersed repeats. The evolution of genomes is characterized by a substantial abundance of repetitive sequences, which play roles highly dependent on specific structural features and modes of multiplication [40]. It has been hypothesized that variation in these repetitive sequences significantly influences mitochondrial (mt) genome rearrangement and evolution. Analysis of SSR composition and distribution frequency identified mononucleotide (83.14%), dinucleotide (13.48%), and trinucleotide (3.37%) repeats as the dominant types, with A/T, AT/TA, and TCT as the most common motifs, respectively (Table S1).

**Figure 2:**
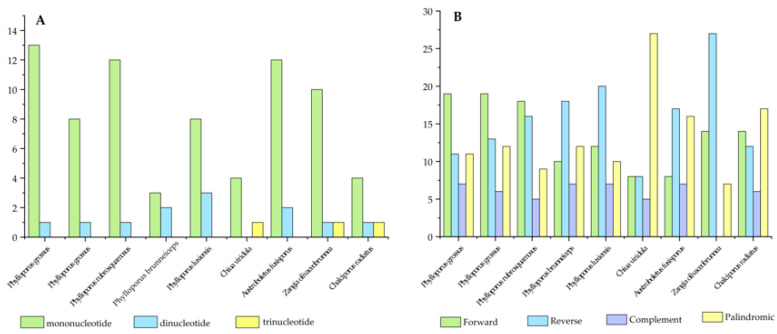
(**A**) Short tandem repeats and (**B**) dispersed repeats in the mitogenomes of the nine Boletaceae specimens.

Simple sequence repeats (SSRs) are widely used as molecular markers in kinship comparison, genetic diversity analysis, variety identification, and breeding research [41]. Analysis of SSRs in the mitogenomes of eight Boletaceae species detected 5 to 14 SSR loci, with lengths ranging from 10 to 72 bp. Mononucleotide, dinucleotide, and trinucleotide repeats were identified as the dominant types: among these, mononucleotide repeats were predominant, accounting for 83.14%, while dinucleotide and trinucleotide repeats accounted for 13.48% and 3.37%, respectively. Their most common motifs were A/T, AT/TA, and TCT, respectively. Notably, no trinucleotide repeats were detected in any of the *Phylloporus* species (Figure 2A).

A total of 86 tandem repeat sequences with a match rate ≥ 95% were identified across the eight species. Among these, *Phylloporus grossus* contained the fewest tandem repeats, with only three, contrasting sharply with *Chalciporus radiatus*, which contained twenty-seven (Table S2).

Dispersed repeat analysis revealed that all *Phylloporus* species, along with most other taxa examined, contained forward (F), reverse (R), complementary (C), and palindromic (P) repeats. Among the species studied, the number of forward repeats was generally similar to that of reverse repeats. In the mitogenomes of *Phylloporus* species, the number of forward repeats was relatively high, ranging from 10 (*Phylloporus brunneiceps*) to 19 (*Phylloporus grossus*). Complementary repeats were the least common type among all species, ranging from 0 to 7 (Table S3). *Zangia olivaceobrunnea* was the only species that lacked complementary repeats (Figure 2B).

### 3.3. Genome Rearrangement Analysis

**Figure 3:**
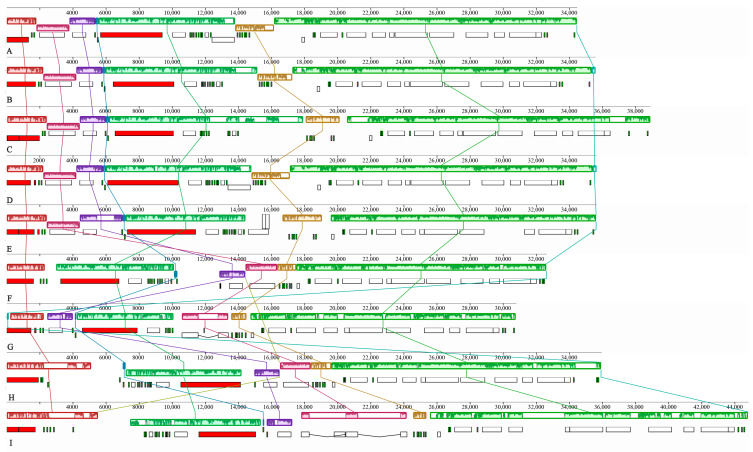
Collinear analysis of mitogenomes in Boletaceae. The colored rectangles beneath the homologous blocks represent the positions and lengths of different types of genes. The same-origin sections are connected by lines of corresponding colors. (**A**) *Phylloporus grossus* HKAS105262; (**B**) *Phylloporus grossus* HKAS105260; (**C**) *Phylloporus brunneiceps* HKAS105261; (**D**) *Phylloporus rubrosquamosus* HKAS105259; (**E**) *Phylloporus luxiensis* HKAS105263; (**F**) *Chiua viridula* HKAS105270; (**G**) *Austroboletus fusisporus* HKAS105269; (**H**) *Zangia olivaceobrunnea* HKAS105268; (**I**) *Chalciporus radiatus* HKAS105264.

Collinear analysis of the mitogenomes of four *Phylloporus* species and four other Boletaceae species revealed the presence of seven homologous blocks, designated A through G, across the nine mitogenomes (Figure 3). The order of these seven homologous regions was completely consistent within the genus *Phylloporus*. In contrast, the arrangement of blocks B, C, and D showed noticeable differences among species from different genera within the Boletaceae family.

The sizes of these seven homologous blocks were not uniform. Block G was the largest of all four *Phylloporus* species, while blocks D and G were relatively conserved. The lengths of the remaining blocks exhibited some degree of variation. Compared to the *Phylloporus* species, the mitogenome of *Chalciporus radiatus* is larger, and its homologous blocks are relatively longer, indicating a positive correlation between the size of the homologous blocks and the overall genome size.

**Figure 4:**
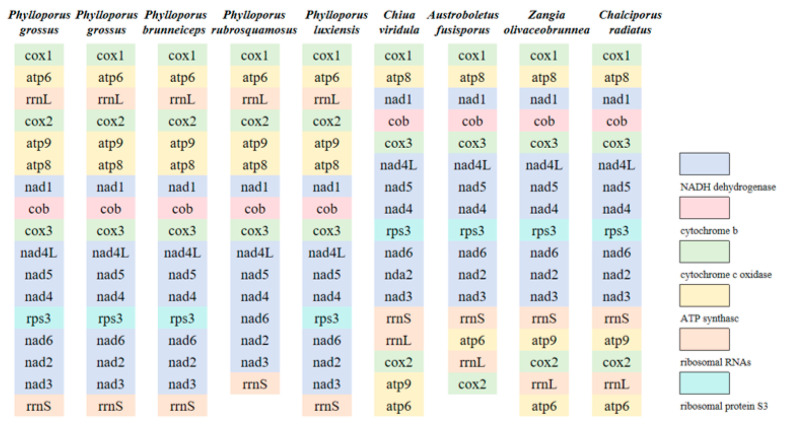
Gene order analysis in nine specimens of Boletaceae collected from Jiangxi Province. Each colored block represents a distinct type of gene. A total of 14–15 PCGs and 2 rRNA genes were used in this analysis.

The gene order is largely consistent among the five *Phylloporus* mitogenomes. However, mitogenome rearrangements were observed among species within the Boletaceae mitogenomes (Figure 4). These rearrangements may be attributed to gene duplication and insertion events.

The gene order between atp8 and rrnS was relatively conserved in the mitogenomes of Boletaceae. Rearrangements were identified in five protein-coding genes (cox1, cox2, atp6, atp9) and two rRNA genes (rrnS, rrnL). Compared to other Boletaceae mitogenomes, two specific gene inversions were observed in the *Phylloporus* mitogenomes: the first involved an inversion between the cox1 and atp6 genes, and the second was an inversion centered around the cox2 gene, involving atp9 and rrnL.

### 3.4. Protein-Coding Genes of the Nine Boletaceae Mitogenomes

In the mitogenomes of the nine Boletaceae species, we found the dominance of ATG as the Start Codon in core protein-coding genes, and TAA is the most common stop codon. Whereas the cox1 gene of *Phylloporus grossus* and *Phylloporus rubrosquamosus* employs TTG as the start codon, and their atp8 gene utilizes TAG as the stop codon.

The cox2 gene of *Chiua viridula* uses TTG as the start codon, while its nad1 and atp8 genes use TAG as the stop codon. The cox1 gene of *Austroboletus fusisporus* uses TTG as the start codon, and its nad3 gene uses TAG as the stop codon. The atp8 gene of *Zangia olivaceobrunnea* uses TAG as the stop codon (Table S4).

**Figure 5:**
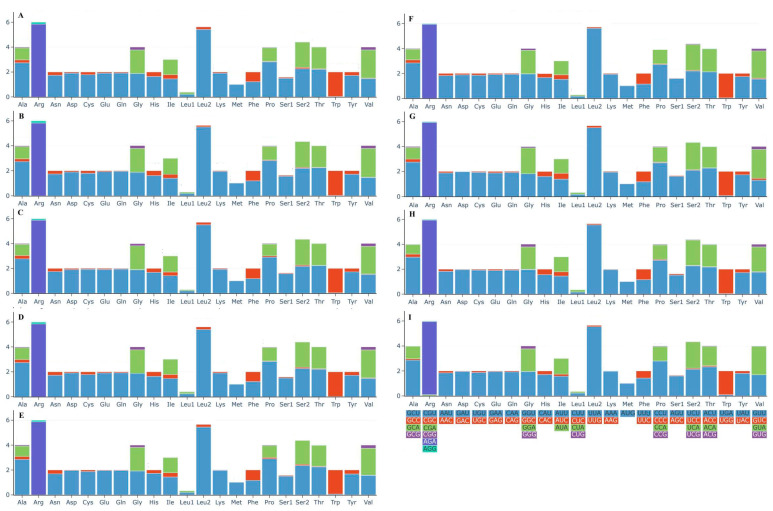
Codon usage in the mitogenomes of the nine Boletaceae specimens. Frequency of codon usage is plotted on the y-axis. Different colors respectively represent the corresponding codons. (**A**) *Phylloporus grossus* HKAS105262; (**B**) *Phylloporus grossus* HKAS105260; (**C**) *Phylloporus brunneiceps* HKAS105261; (**D**) *Phylloporus rubrosquamosus* HKAS105259; (**E**) *Phylloporus luxiensis* HKAS105263; (**F**) *Chiua viridula* HKAS105270; (**G**) *Austroboletus fusisporus* HKAS105269; (**H**) *Zangia olivaceobrunnea* HKAS105268; (**I**) *Chalciporus radiatus* HKAS105264.

Analysis of codon usage and relative synonymous codon usage (RSCU) in the nine mitogenomes showed that they are highly similar in codon usage patterns, but there are significant differences in the RSCU of each amino acid (Figure 5). Among them, AGA (for glutamine; Arg) and UUA (for cysteine; Leu2) are the most frequently used codons, and these two amino acids are also the most abundant. In contrast, Met (methionine) is only encoded by AUG, with a low usage frequency of only 2.15%. Additionally, among the 62 analyzed codons, 27 have an RSCU value greater than 1.0.

### 3.5. Analysis of the tRNA Secondary Structure in Phylloporus

**Figure 6:**
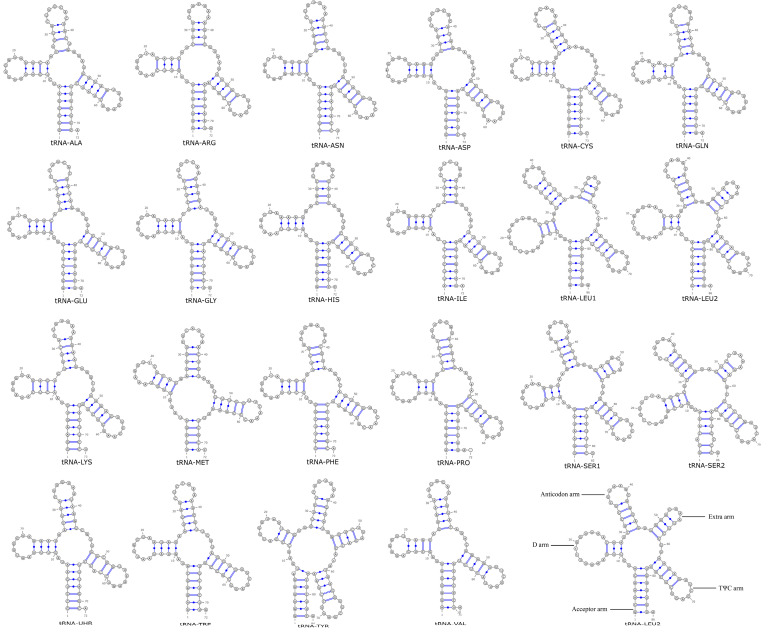
Structure prediction of mitochondrial genome tRNA of *Phylloporus luxiensis*.

In the mitochondrial genome of *Phylloporus*, there are 24 tRNA genes, which are capable of transporting all 20 amino acids. Among them, the secondary structures of tRNA-Leu, tRNA-Ser, and tRNA-Tyr possess one extra loop, while the rest exhibit the typical cloverleaf structure (Figure 6). Among the four *Phylloporus* species, the mitochondrial genome tRNA genes contain 2 to 4 base mismatches, with *Phylloporus brunneiceps* and *Phylloporus rubrosquamosus* having the fewest base mismatches. Compared to other species in the Boletaceae family, the tRNA-Leu of *Phylloporus* has an additional base G in the D-loop. *Austroboletus fusisporus* shows the highest number of base mismatches compared to *Phylloporus*, with 6 mismatches. In all Boletaceae species studied, the secondary structures of tRNA-Leu, tRNA-Ser, and tRNA-Tyr feature one extra loop (Figures S1–S8).

### 3.6. Evolutionary Rates of Core Genes in Boletaceae

**Figure 7:**
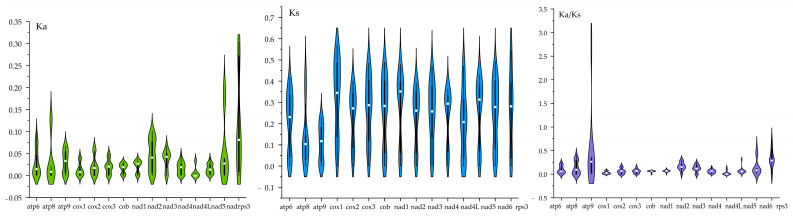
Evolutionary rates of genes in Boletaceae species. Ka represents the nonsynonymous substitution rate per nonsynonymous site, and Ks denotes the synonymous substitution rate per synonymous site.

To further investigate the evolutionary dynamics of Boletaceae, selection pressure analysis was conducted on protein-coding genes (PCGs) at the amino acid level. Due to the skewed distribution of the data, the median was used to represent the overall trend (Figure 7).

Among the 15 PCGs analyzed, the rps3 gene exhibited the highest nonsynonymous substitution rate (Ka), while the atp9 gene showed the lowest Ka value. Overall, all 15 genes had Ka values below 0.35. In terms of synonymous substitution rates (Ks), cox1 displayed the highest value, whereas atp8 had the lowest. All Ks values remained under 0.7.

The majority of core PCGs across Boletaceae species exhibited Ka/Ks ratios less than 1, indicating the prevalence of purifying selection at the amino acid level. Notably, nad4L and cox1 displayed significantly low Ka/Ks values, reflecting stronger purifying selection pressures on these genes.

### 3.7. Phylogenetic Relationships

Based on specimens of eight species from Jiangxi Province and mitogenome data of 19 related species (17 Boletaceae, 2 Paxillaceae) retrieved from NCBI [28,29,36,37,38,39,42], 15 core mitochondrial protein-coding genes (PCGs) were extracted (Table 1). Individual gene matrices were constructed and concatenated for phylogenetic analysis. With Paxillaceae designated as the outgroup, maximum likelihood (ML) and Bayesian inference (BI) methods were used to reconstruct phylogenetic trees.

**Figure 8:**
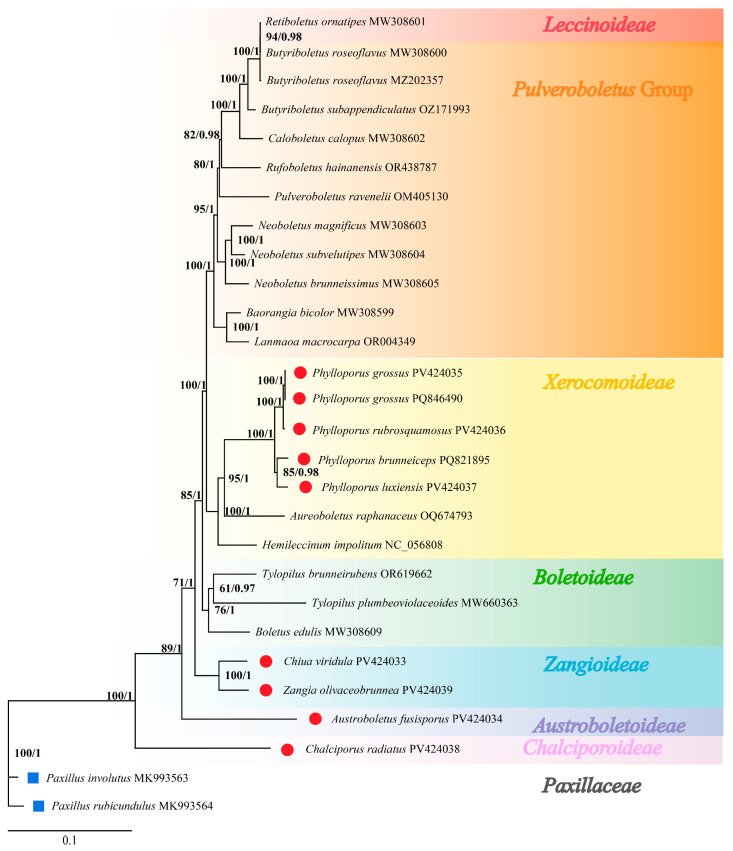
Phylogenetic tree constructed based on Bayesian inference (BI) and maximum likelihood (ML) analyses of 15 core protein-coding genes (atp6, atp8, atp9, cob, cox1, cox2, cox3, nad1, nad2, nad3, nad4, nad4L, nad5, nad6, and rps3). RAxML likelihood bootstrap support values (98%) and Bayesian posterior probabilities (PP > 0.71) are indicated above or below the branches as BS/PP. The red circles mark the newly sequenced species, and the blue rectangles indicate the outgroups.

The resulting ML and BI trees exhibited identical topology. For clarity, only the ML tree is presented (Figure 8), with BI posterior probabilities indicated at corresponding nodes.

The phylogeny supported seven distinct major clades within Boletaceae, each confirming the monophyly of the corresponding subfamily. This structure aligns with the recently proposed classification system of seven subfamilies: *Austroboletoideae*, *Boletoideae*, *Chalciporoideae*, *Leccinoideae*, *Xerocomoideae*, *Zangioideae*, and the *Pulveroboletus* Group [43,44]. The genus *Phylloporus* was clustered within the Xerocomoideae subfamily and showed the closest affinity to the Boletoideae subfamily. There are subclades within the genus; one clade includes *Phylloporus grossus* and *Phylloporus rubrosquamosus*, the other clade includes *Phylloporus brunneiceps* and *Phylloporus luxiensis*. The phylogenetic relationships revealed in this study are consistent with prior studies based on 28S, *TEF1*, *RPB1*, and *RPB2* molecular markers [44].

## 4. Discussion

Accurate taxonomic identification is a prerequisite for the efficient utilization and safe development of fungal resources within the Boletaceae family. *Phylloporus* species are readily identified at the genus level and belong to the subfamily Boletoideae, yet species delimitation based on morphology remains difficult due to ambiguous boundaries. Recent advances in molecular phylogenetics have led to the reevaluation of several taxa within the genus and the description of new species, greatly improving our understanding of its diversity in China [45,46,47]. As of 2024, 70 toxic fungal species have been documented in China within the order Boletales [48]. Interspecific morphological differences in Boletaceae are often subtle and overlapping, making species classification and identification based solely on morphological characteristics challenging [49].

Although nuclear genomes and molecular markers are widely used in fungal identification [50], mitogenomes have emerged as a powerful tool for phylogenetic studies due to their relatively small size, ease of acquisition, and substantially greater information content compared to single molecular markers [8]. The evolutionary dynamics of mitochondrial genomes provide rich insights into the history and diversity of life on Earth [51]. Understanding these dynamics offers critical context for interpreting phylogenetic relationships, elucidating species divergence, and unravelling the molecular mechanisms underpinning adaptation and speciation [52]. At present, the available mitogenome data for fungi remain very limited [28]. There are some thirty sequenced mitochondrial genomes in the family Boletaceae, while the mitochondrial genomes of *Phylloporus* have not been reported.

This study presents the first complete mitogenomes of eight species from seven subfamilies of Boletaceae in Jiangxi Province. The genomes were circular, ranging from 30,801 bp to 44,823 bp, consistent with the documented size range for this family [28]. Comparative analysis reveals that the genus *Phylloporus* possesses a distinctive mitogenomic architecture, characterized by a combination of a relatively conserved gene order, a moderate genome size, and notable variations in intergenic spacer length and repetitive element content.

DNA base composition is a fundamental genomic trait, with variations influencing species distribution, ecological adaptation, and life-history strategies [53]. We comparatively analyzed the genomic characteristics of 24 species in the Boletaceae family and 2 species in the *Paxillus* genus (Table 3). Among the species examined, ranging from 30,801 bp to 44,823 bp, and GC content ranges from 21.03% to 24.49%. Species within *Phylloporus* exhibit notably higher GC content compared to other groups in the Boletaceae family. Elevated GC content may increase mutation rates [54], which could be associated with the distinctive lamellate hymenophore structure in *Phylloporus*. However, this potential relationship is a tentative hypothesis that requires further research to verify. Crucially, the factors leading to differences in gene size are the accumulation of intron and repeat-driven changes.

When compared to other genera within the Boletaceae, this profile of *Phylloporus* stands in clear contrast. For instance, in this study, the mitogenome of *Chalciporus radiatus* is the largest, and it also contains the most tandem repeat sequences, indicating that repetitive elements play a certain role in genomic expansion across the family. Conversely, *Austroboletus fusisporus* maintains the smallest genome despite harboring a group I intron, underscoring that intronic regions are not the predominant driver of size in these lineages and further highlighting the unique *Phylloporus* model where repetitive elements, not introns, modulate size. However, due to the presence of introns, the mitochondrial genome size of species in the Boletaceae is much smaller than that of the *Suillus* genus [55]. This comparative analysis confirmed that the accumulation of repetitive sequences and the number of introns is common drivers of mitochondrial genome size variation in Boletaceae species.

Repetitive sequences play a crucial role in genomic recombination and restructuring [56], and their accumulation can lead to genomic expansion or contraction over evolutionary time [57,58]. The consistency in SSR types across Boletaceae mitogenomes suggests a high degree of sequence conservation within the family. Boletaceae species have fewer SSR types than plants, and hexameric SSRs are present in *Hydrangea chinensis* [59]. The absence of trinucleotide repeats in *Phylloporus* may be attributed to mitochondrial genomic characteristics and shorter sequence lengths. Recombination events have been shown to play a crucial role in genome evolution and adaptation [60]. The presence of a large number of SSRs further highlights the dynamic nature of the mitochondrial genome. It is known that SSRs can lead to genomic instability and variability, which may in turn affect gene expression and mitochondrial function [61].

Although mitochondrial genes are generally conserved due to their essential roles in cellular metabolism, gene order in fungal mitochondria can vary considerably. The accumulation of repetitive DNA elements in intergenic regions is a key driver of mitochondrial gene rearrangements. Frequent rearrangements have been reported in the genus Boletus [28], suggesting divergent evolutionary trajectories among lineages. Gene order analysis here reveals significant intergeneric variation in mitogenomes within Boletaceae, whereas *Phylloporus* exhibits high intragenomic conservation, possibly due to intramolecular recombination in the ancestral Basidiomycete mitogenome [62]. This contrast between intrageneric stability and intergeneric plasticity supports the utility of mitochondrial gene rearrangements as taxonomic markers.

Codon usage bias is critical for protein function and translational accuracy and can provide insights into evolutionary and environmental adaptation mechanisms [63]. In this study, codon preference patterns are highly conserved across Boletaceae mitogenomes, though minor differences exist in the start and stop codons of core PCGs. Among the 62 codons analyzed, 27 have RSCU values > 1.0, a pattern potentially shaped by long-term environmental stress.

The Ka/Ks ratios for all 15 core PCGs are below 1, indicating dominance of purifying selection across the family. However, based on qualitative observations, the atp9 gene shows signs of positive selection in *Phylloporus luxiensis*, *Phylloporus rubrosquamosus*, and *Phylloporus grossus*, possibly reflecting ecological adaptation. Significantly lower Ka/Ks values for nad4L and cox1 suggest intensified purifying selection, which may reduce genetic diversity and distort site frequency spectra—effects that could mimic population expansion or positive selection [64]. These differential selection pressures provide new insights into the adaptive evolution of functional genes during the diversification of *Phylloporus*.

Phylogenetic tree (Figure 7) confirmed seven major subfamilial clades within Boletaceae [44]. Our phylogenetic analysis groups *Phylloporus brunneiceps* with *P. luxiensis*, and *P. rubrosquamosus* with *P. grossus*. This is consistent with the nuclear gene-based phylogeny by Zeng et al. [1], where *P. brunneiceps* and *P. luxiensis* formed part of Clade I, while *P. rubrosquamosus* fell within Clade VI. The phylogenetic tree we reconstructed based on ITS sequences also confirms this (Figure S9).

Nonetheless, compared to topologies inferred from nuclear gene fragments, mitochondrial-based trees show slight differences in branching patterns and subfamilial placements, possibly due to the autonomous replication, transcription, and translation of mitogenomes, their lack of recombination, and maternal inheritance [8]. Alternatively, limited taxonomic sampling may affect clustering accuracy. These findings demonstrate that concatenated mitochondrial gene sets can accurately recover deep evolutionary relationships, highlighting their value as a robust tool for phylogenetic inference in Basidiomycetes, and providing a reliable basis for taxonomic revision, species identification, and conservation planning in Boletaceae.

The evolution of the mitochondrial genome of *Phylloporus*, an ectomycorrhizal fungus, may be the result of a balance between functional conservation and ecological adaptability. The purification and selection of core energy metabolism genes ensure their basic symbiotic functions, while the adaptive mutations of local genes and the plasticity of genomic structure promote the adaptation of fungi to hosts and the environment. Ultimately, through the optimization of the symbiotic system between fungi and hosts, the stability of the forest ecosystem is maintained.

## 5. Conclusions

*Phylloporus* is an important ectomycorrhizal and edible fungus, yet its molecular data are scarce. Mitochondrial genomes are valuable for phylogeny and species differentiation due to their high copy number and ease of acquisition. While many Boletales mitogenomes have been published, none are available for the genus *Phylloporus*. In this study, we presented the first complete mitochondrial genomes of four *Phylloporus* species from Jiangxi Province, China, along with four additional Boletaceae species, providing the first mitogenomic insights into the genus *Phylloporus*. Our results revealed that the mitogenomes of *Phylloporus* are exhibiting a relatively conserved gene order and content yet are showing notable variations in repetitive sequences and intergenic regions. Notably, *Phylloporus* mitogenomes lacked trinucleotide SSRs and contained few introns, suggesting that repetitive elements rather than intronic expansions are the primary drivers of genome size variation within this genus.

Comparative genomic analyses highlighted both shared and distinct evolutionary features between *Phylloporus* and other Boletaceae genera. While codon usage bias was largely conserved across the family, gene order and repetitive element profiles served as informative taxonomic markers. Phylogenetic reconstruction based on 15 core PCGs strongly supported the monophyly of *Phylloporus* and its placement within the subfamily Xerocomoideae, consistent with previous nuclear-based studies. Furthermore, the Ka/Ks ratios indicated that most PCGs are under purifying selection, possibly reflecting ecological adaptation. These characteristics represent not only molecular signatures of evolutionary divergence but also reflect long-term adaptive strategies to subtropical forest ecosystems, offering new perspectives on the evolution of ectomycorrhizal fungi.

This study not only enriches the mitochondrial genomic resources for Boletaceae but also demonstrates the utility of mitogenomes in resolving phylogenetic relationships and understanding genomic evolution in ectomycorrhizal fungi. The distinct mitogenomic architecture of *Phylloporus* provides a valuable model for studying fungal adaptation and diversification. Future efforts should focus on expanding mitogenome sequencing to include more taxonomically diverse and morphologically cryptic taxa within Basidiomycota to further elucidate the evolutionary history and functional genomics of these ecologically and economically significant fungi.

## Figures and Tables

**Table 1:** Information of samples for phylogenetic analysis.

Family		Species	Genbank Number	Size (bp)	References
Boletaceae		*Aureoboletus raphanaceus*	NC079662	42,157	[36]
		*Baorangia bicolor*	MW308599	38,082	[28]
		*Boletus edulis*	MW308609	34,763	[28]
		*Butyriboletus subappendiculatus*	OZ171993	35,023	Unpublished
		*Butyriboletus roseoflavus*	MW308600	36,622	[28]
		*Butyriboletus roseoflavus*	MZ202357	36,551	Unpublished
		*Caloboletus calopus*	MW308602	32,883	[28]
		*Hemileccinum impolitum*	NC056808	39,362	Unpublished
		*Lanmaoa macrocarpa*	NC080885	38,139	[30]
		*Neoboletus brunneissimus*	MW308605	42,147	[28]
		*Neoboletus magnificus*	MW308603	39,449	[28]
		*Neoboletus obscureumbrinus*	MW308607	39,929	[28]
		*Pulveroboletus ravenelii*	NC061666	43,528	[37]
		*Rufoboletus hainanensis*	OR438787	36,592	[38]
		*Retiboletus ornatipes*	MW308601	36,785	[28]
		*Tylopilus brunneirubens*	NC084291	32,389	[39]
		*Tylopilus plumbeoviolaceoides*	NC056835	37,242	[29]
Paxillaceae		*Paxillus involutus*	NC045203	39,109	[27]
		*Paxillus rubicundulus*	NC045204	41,061	[27]

**Table 2:** The nine newly sequenced mitochondrial genomes of Boletaceae in Jiangxi Province.

Species Name	Genbank Number	Length(bp)	GCRate(%)	AT-Skew	GC-Skew	tRNAs	Introns	Number of Repeats
*Austroboletus fusisporus*	PV424034	30,801	24.05	−0.03	0.01	20	1	5
*Chalciporus radiatus*	PV424038	44,823	21.69	−0.02	−0.02	24	0	27
*Chiua viridula*	PV424033	32,641	23.68	0.06	−0.02	25	0	15
*Phylloporus brunneiceps*	PQ821895	38,908	24.44	0.04	−0.04	28	0	6
*Phylloporus grossus*	PQ846490	35,591	24.24	0.06	−0.03	24	0	3
*Phylloporus grossus*	PV424035	35,117	24.42	0.05	−0.03	24	0	3
*Phylloporus luxiensis*	PV424037	35,648	24.49	0.06	−0.03	24	1	16
*Phylloporus rubrosquamosus*	PV424036	35,647	24.38	0.05	−0.03	24	1	4
*Zangia olivaceobrunnea*	PV424039	35,949	23.78	−0.02	−0.03	26	0	7

**Table 3:** Comparative genomic characteristics of 24 species o (22 Boletaceae, 2 Paxillaceae).

Species Name	Genbank Number	Length (bp)	GC Rate (%)	AT-Skew	GC-Skew	Number of Repeats
*Austroboletus fusisporus*	PV424034	30,801	24.05	−0.03	0.01	5
*Chalciporus radiatus*	PV424038	44,823	21.69	−0.02	−0.02	27
*Chiua viridula*	PV424033	32,641	23.68	0.06	−0.02	15
*Phylloporus brunneiceps*	PQ821895	38,908	24.44	0.04	−0.04	6
*Phylloporus grossus*	PQ846490	35,591	24.24	0.06	−0.03	3
*Phylloporus grossus*	PV424035	35,117	24.42	0.05	−0.03	3
*Phylloporus luxiensis*	PV424037	35,648	24.49	0.06	−0.03	16
*Phylloporus rubrosquamosus*	PV424036	35,647	24.38	0.05	−0.03	4
*Zangia olivaceobrunnea*	PV424039	35,949	23.78	−0.02	−0.03	7
*Aureoboletus raphanaceus*	NC079662	42,157	22.73	0.03	0.03	8
*Baorangia bicolor*	MW308599	38,082	23.61	0.02	0.01	12
*Boletus edulis*	MW308609	34,763	23.83	0.02	−0.06	12
*Butyriboletus subappendiculatus*	OZ171993	35,023	22.82	0.03	0.02	13
*Butyriboletus roseoflavus*	MW308600	36,622	23.13	0.03	0.02	14
*Butyriboletus roseoflavus*	MZ202357	36,551	23.13	0.03	0.02	12
*Caloboletus calopus*	MW308602	32,883	23.21	0.03	0.02	17
*Hemileccinum impolitum*	NC056808	39,362	23.98	0.02	0.01	5
*Lanmaoa macrocarpa*	NC080885	38,139	23.5	0.03	0.03	14
*Neoboletus brunneissimus*	MW308605	42,147	22.96	0.03	0.02	14
*Neoboletus magnificus*	MW308603	39,449	22.93	0.03	0.02	11
*Neoboletus obscureumbrinus*	MW308607	39,929	23.1	0.03	0.04	9
*Pulveroboletus ravenelii*	NC061666	43,528	23.35	−0.02	0.05	12
*Rufoboletus hainanensis*	OR438787	36,592	23.95	−0.04	−0.02	5
*Retiboletus ornatipes*	MW308601	36,785	23.19	0.03	0.01	13
*Tylopilus brunneirubens*	NC084291	32,389	23.8	−0.02	0.05	9
*Tylopilus plumbeoviolaceoides*	NC056835	37,242	23.02	0.02	0.01	9
*Paxillus involutus*	NC045203	39,109	21.75	−0.09	0.06	53
*Paxillus rubicundulus*	NC045204	41,061	21.03	−0.09	0.05	47

## Data Availability

The original contributions presented in this study are included in the article/Supplementary Materials. Further inquiries can be directed to the corresponding authors.

## Supplementary Materials

The following supporting information can be downloaded at: https://www.mdpi.com/article/10.3390/jof11120831/s1. Table S1: Short tandem repeats. Table S2: tandem repeats. Table S3: dispersed repeats. Table S4: Codon usage. Figure S1: *Austroboletus fusisporus*. Figure S2: *Chalciporus radiatus*. Figure S3: *Chiua viridula*. Figure S4: *Phylloporus rubrosquamosus*. Figure S5: *Phylloprous grossus*. Figure S6: *Phylloprus grossus* 1301. Figure S7: *Phylloprus brunneiceps*. Figure S8: *Zangia olivaceobrunnea.* Figure S9: Phylogenetic analysis of Phylloporus.
