# Supplementary material for: Unveiling the Unique Mitogenome Structure of Phylloporus: Implications for Phylogeny and Evolution in Boletaceae
Source: J Fungi (Basel). 2025 Nov 25;11(12):831. doi: 10.3390/jof11120831 (PMC12733429; doi:10.3390/jof11120831)
Supplement: Supplementary file 1 [file jof-11-00831-s001.zip › FIG6-Structure prediction/Figure S1-Austroboletus fusisporus.pdf]

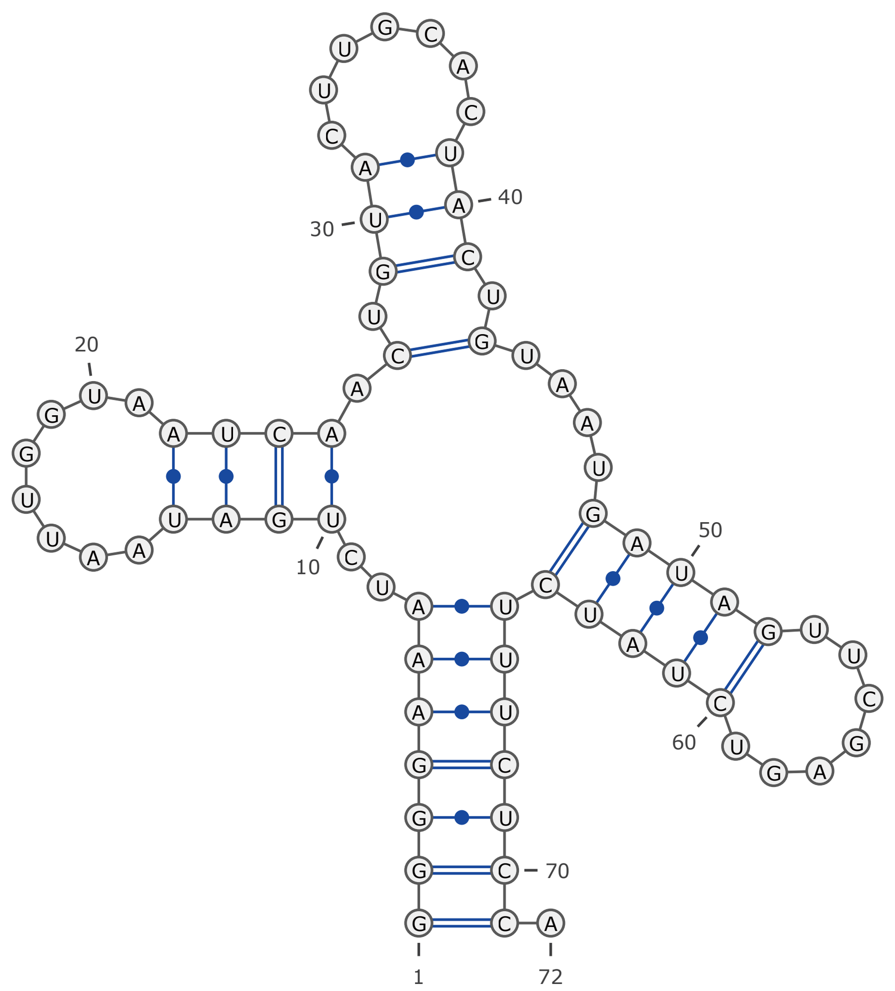

tRNA-ALA

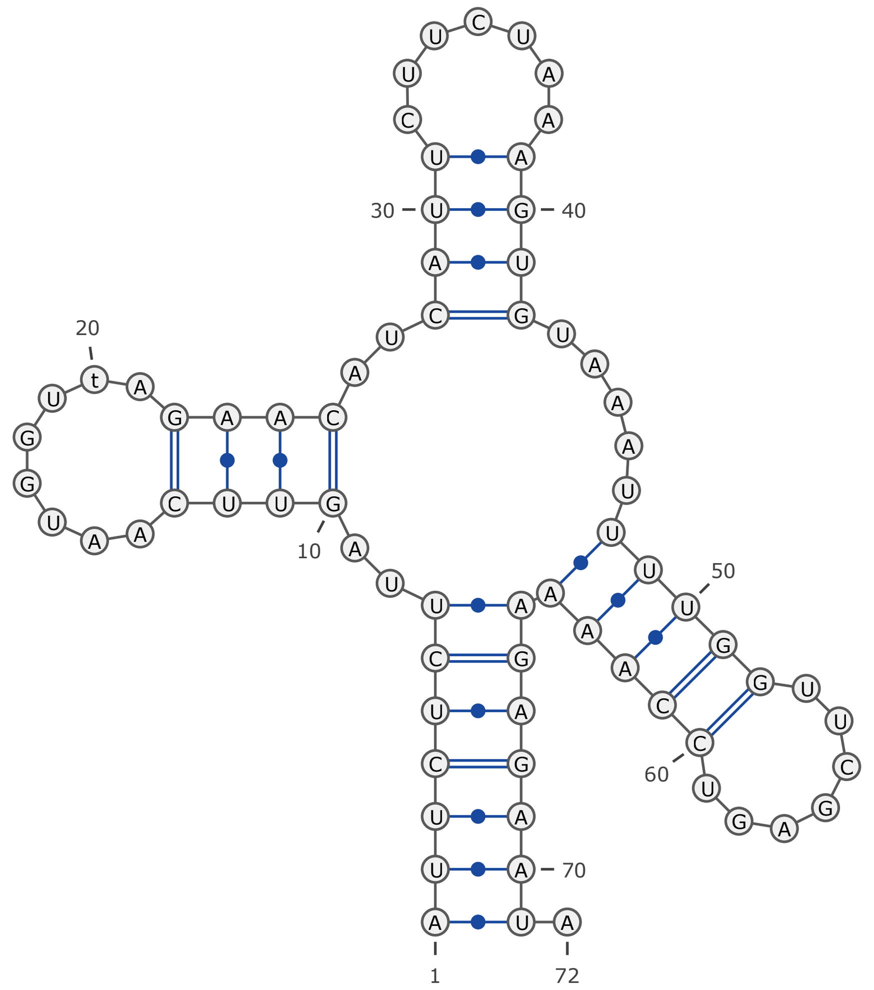

tRNA-ARG

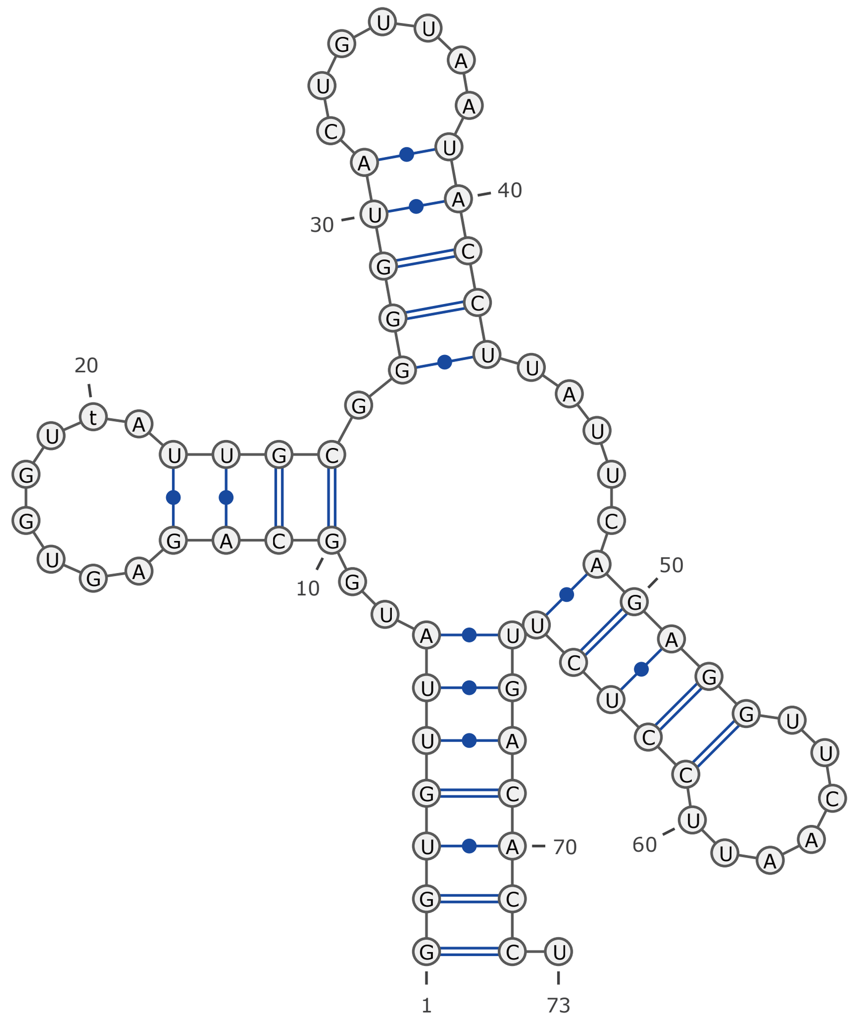

tRNA-ASN

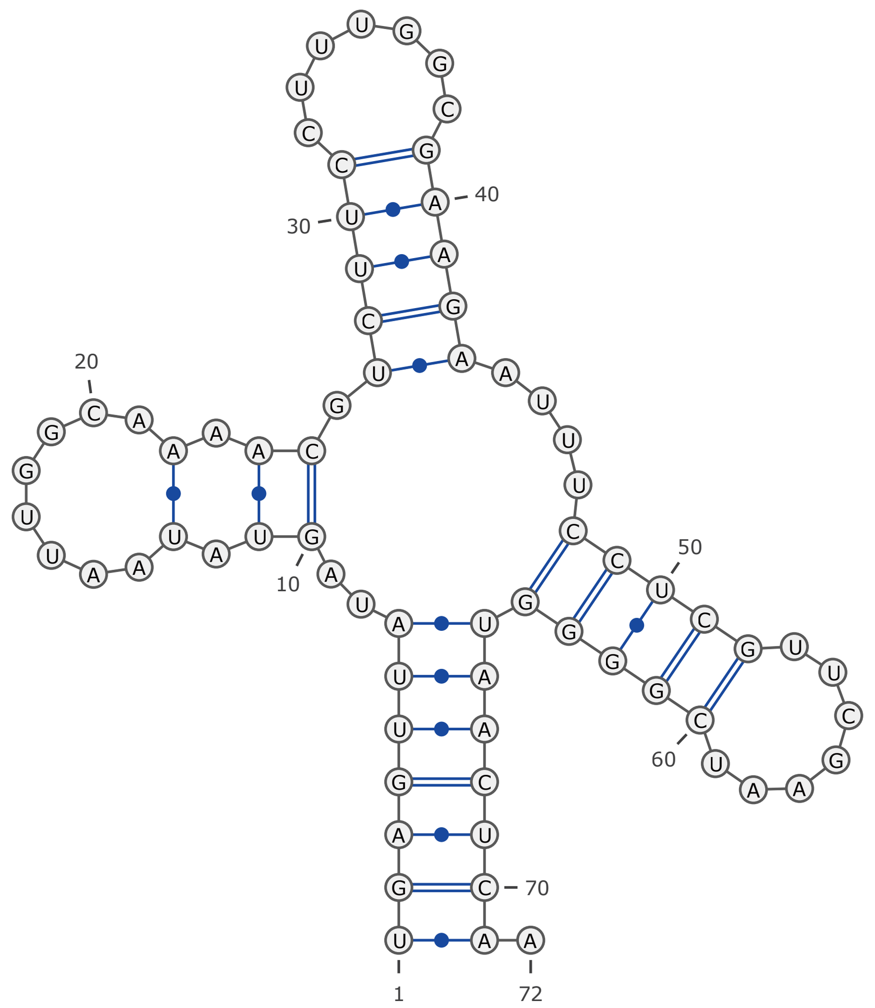

tRNA-GLN

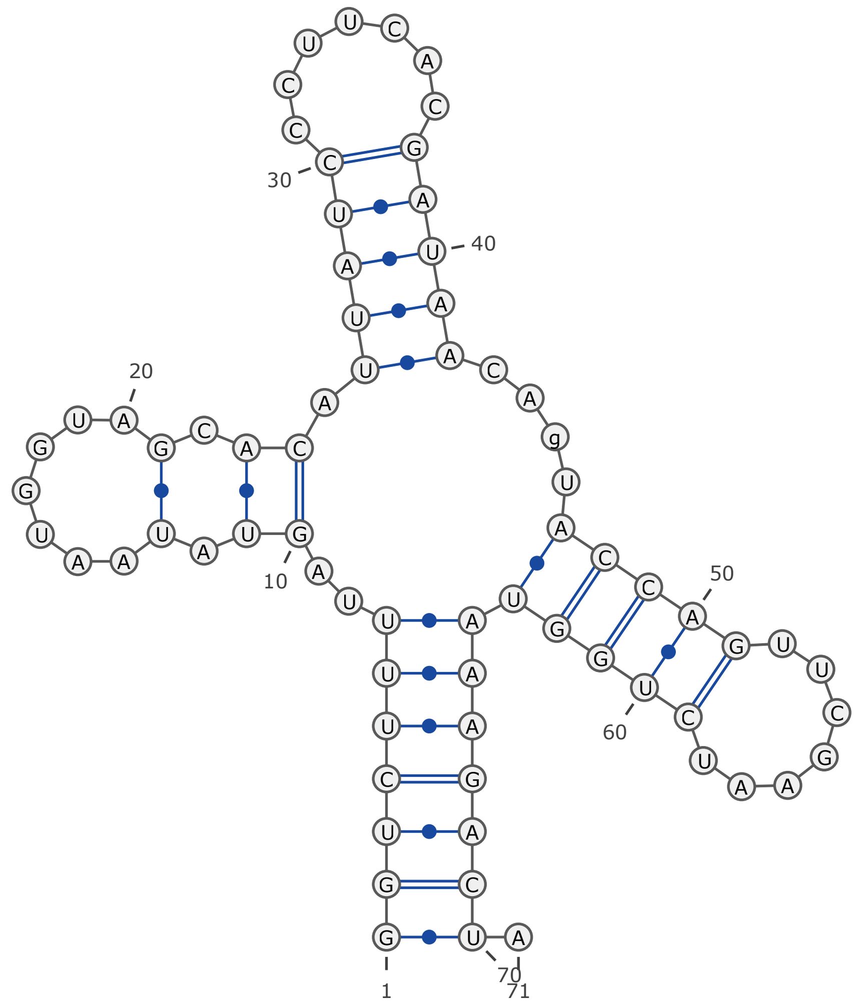

tRNA-GLU

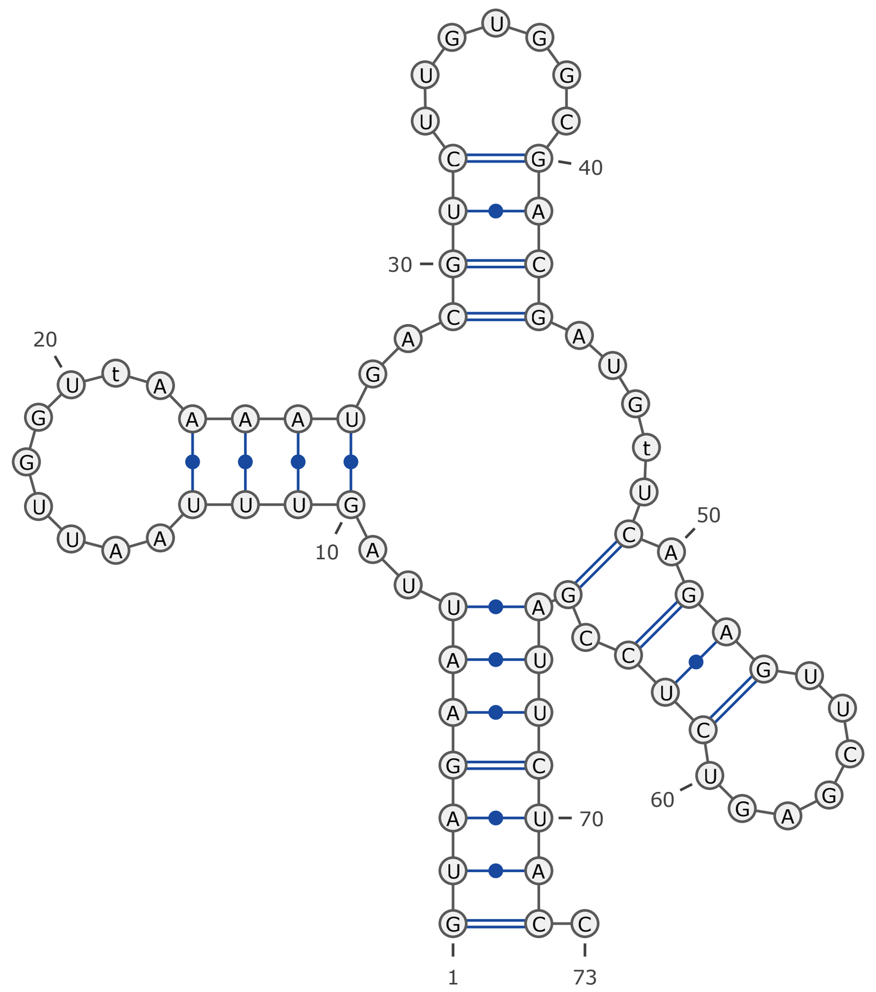

tRNA-HIS

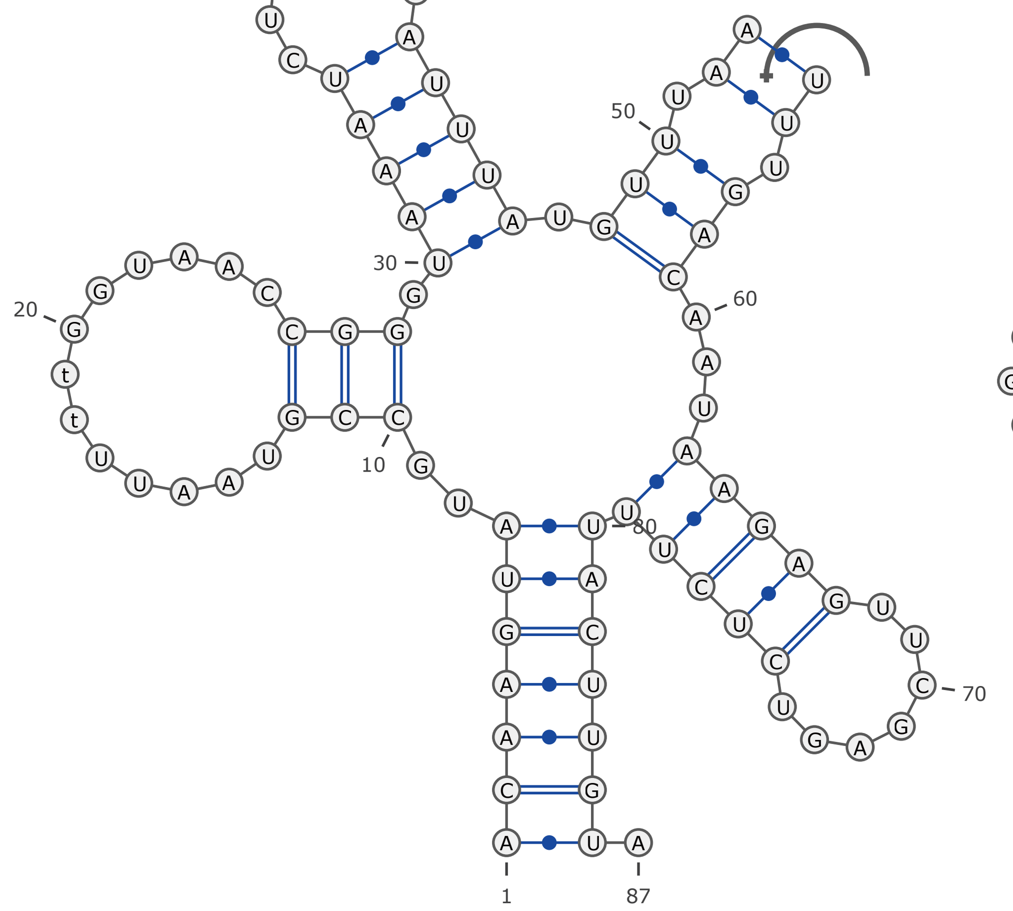

tRNA-LEU

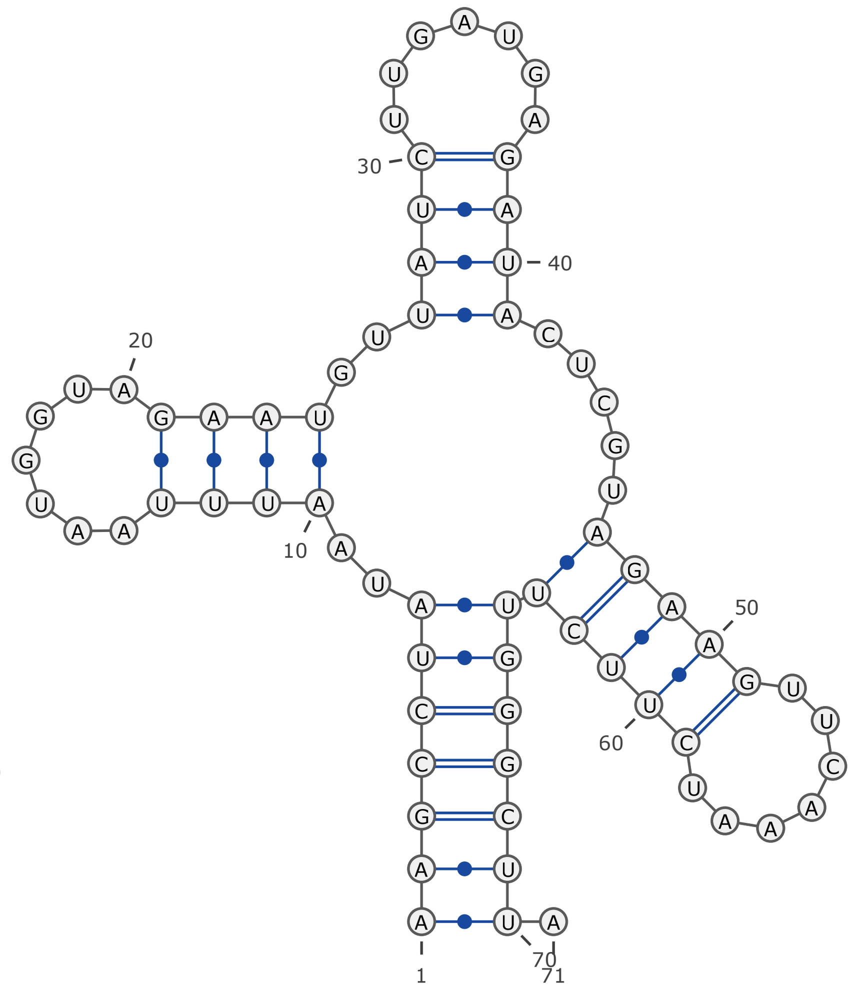

tRNA-LLE

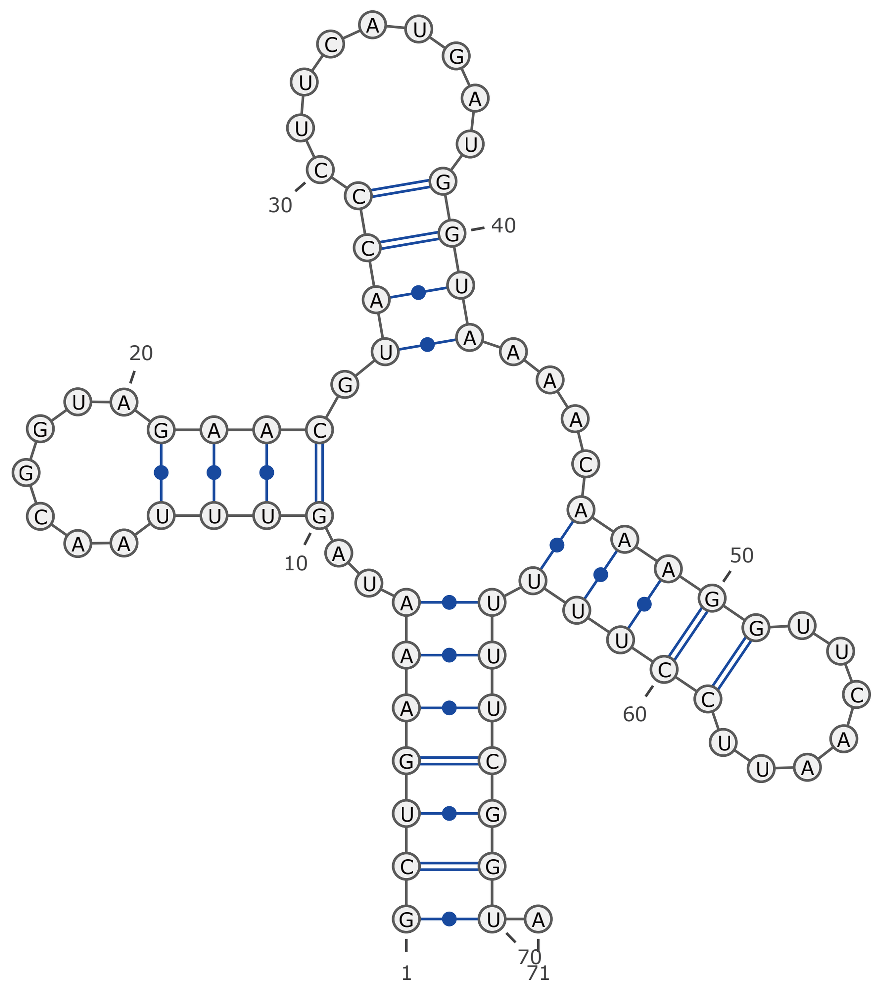

tRNA-MET

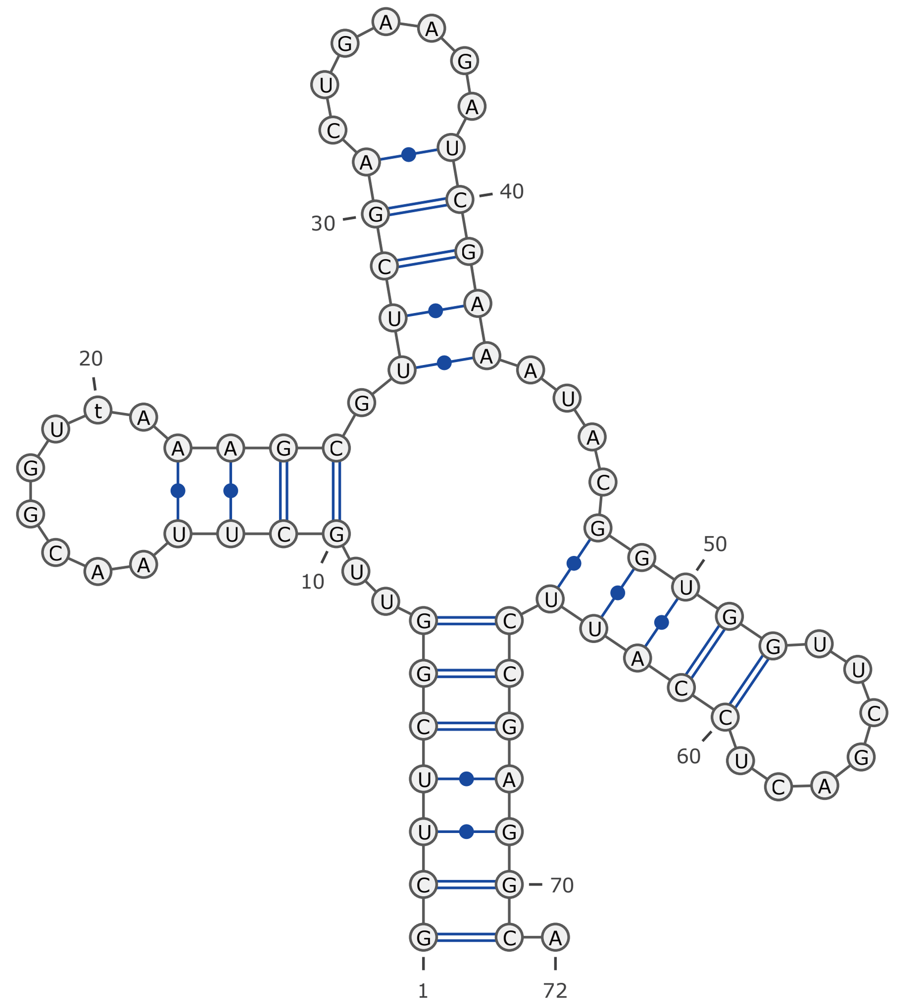

tRNA-PHE

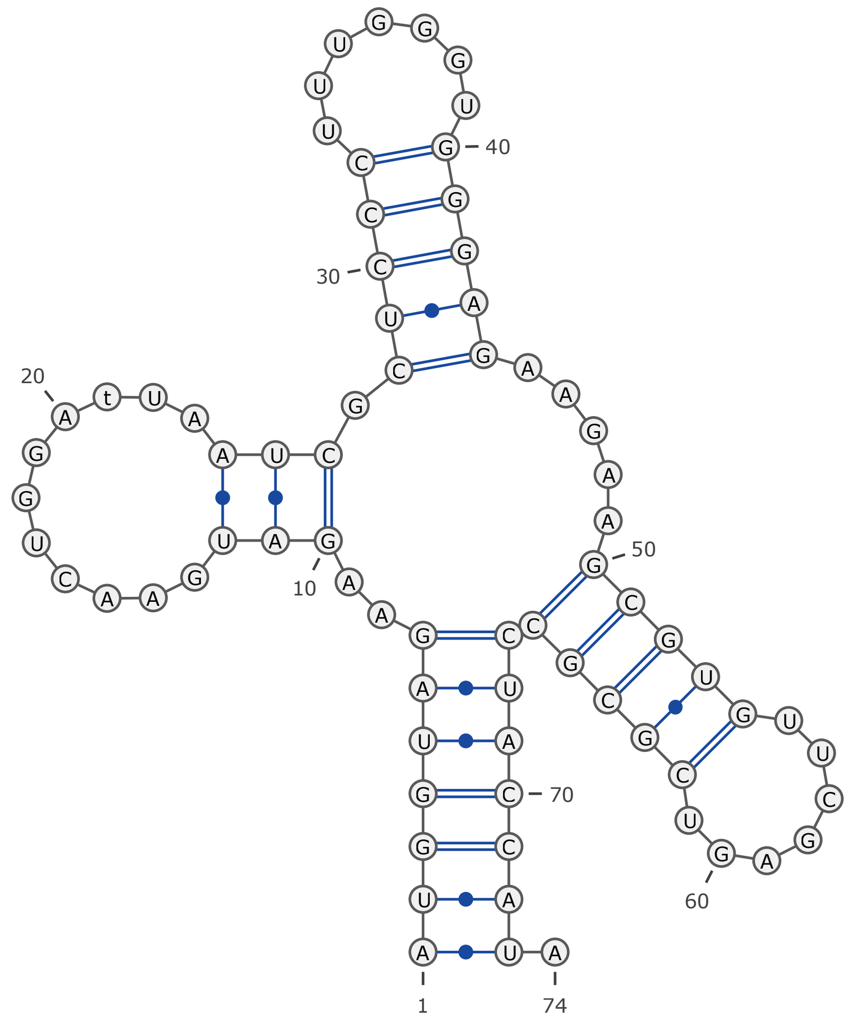

tRNA-PRO

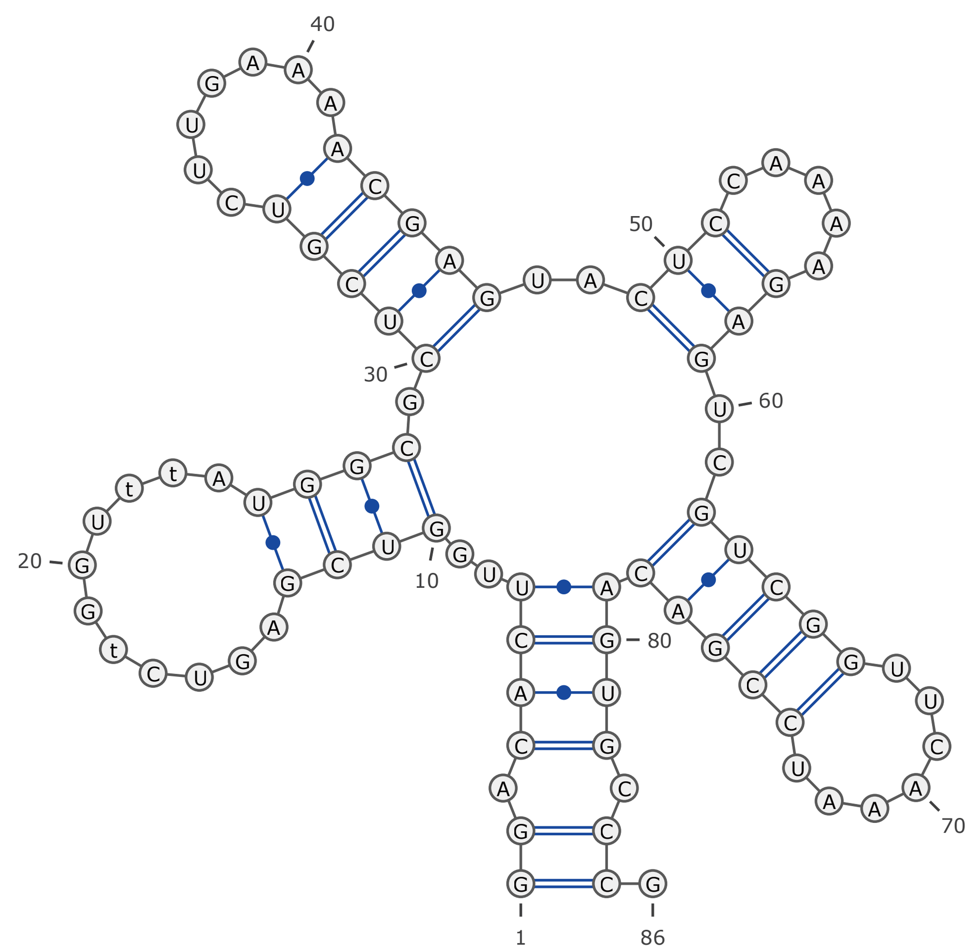

tRNA-SER

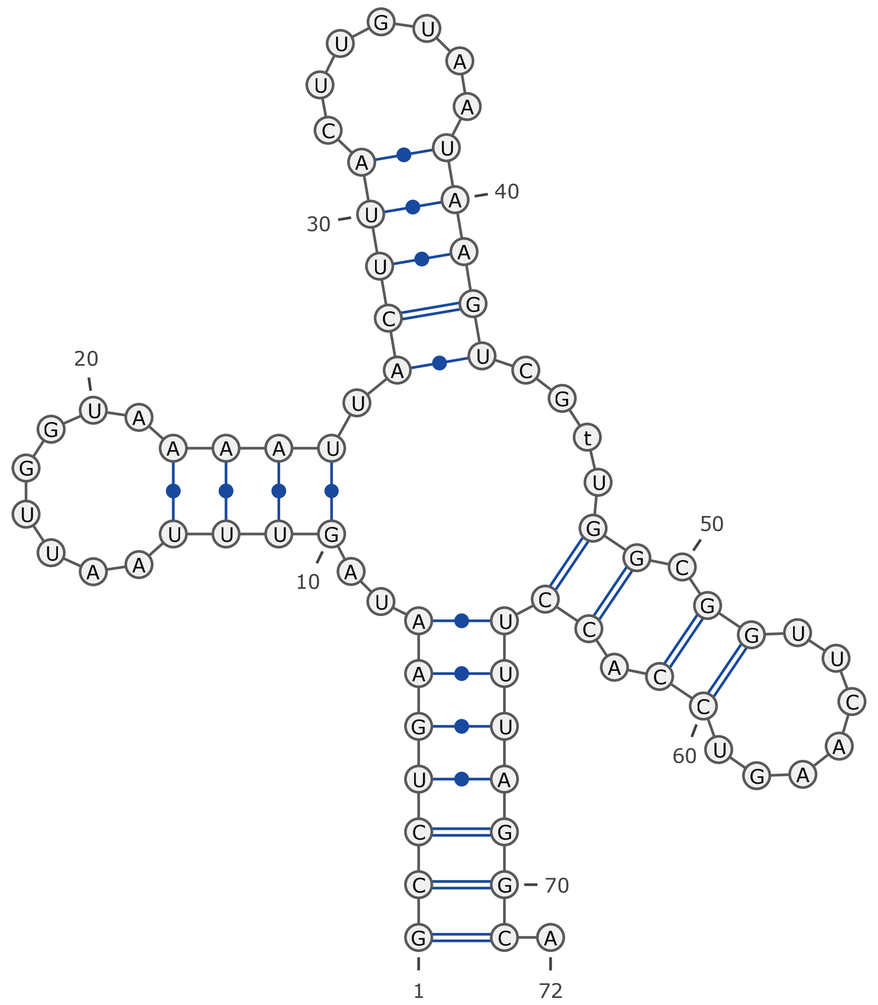

tRNA-THR

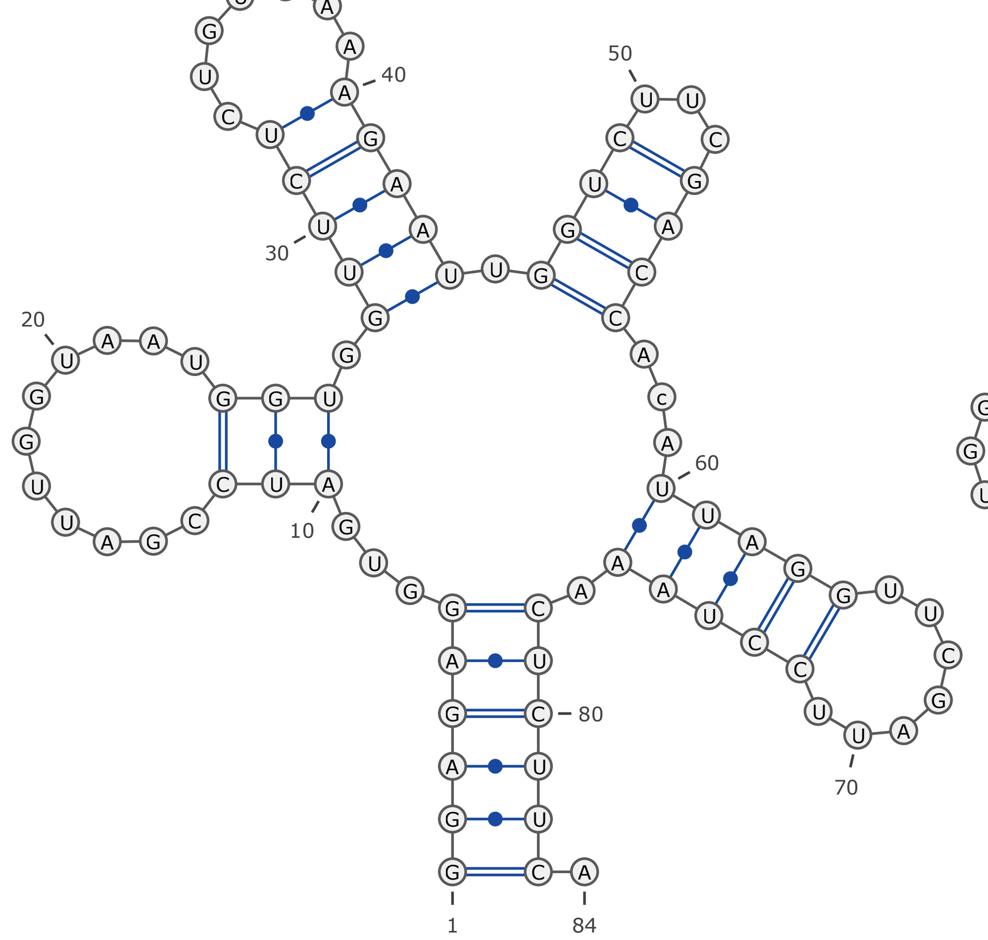

tRNA-TYR

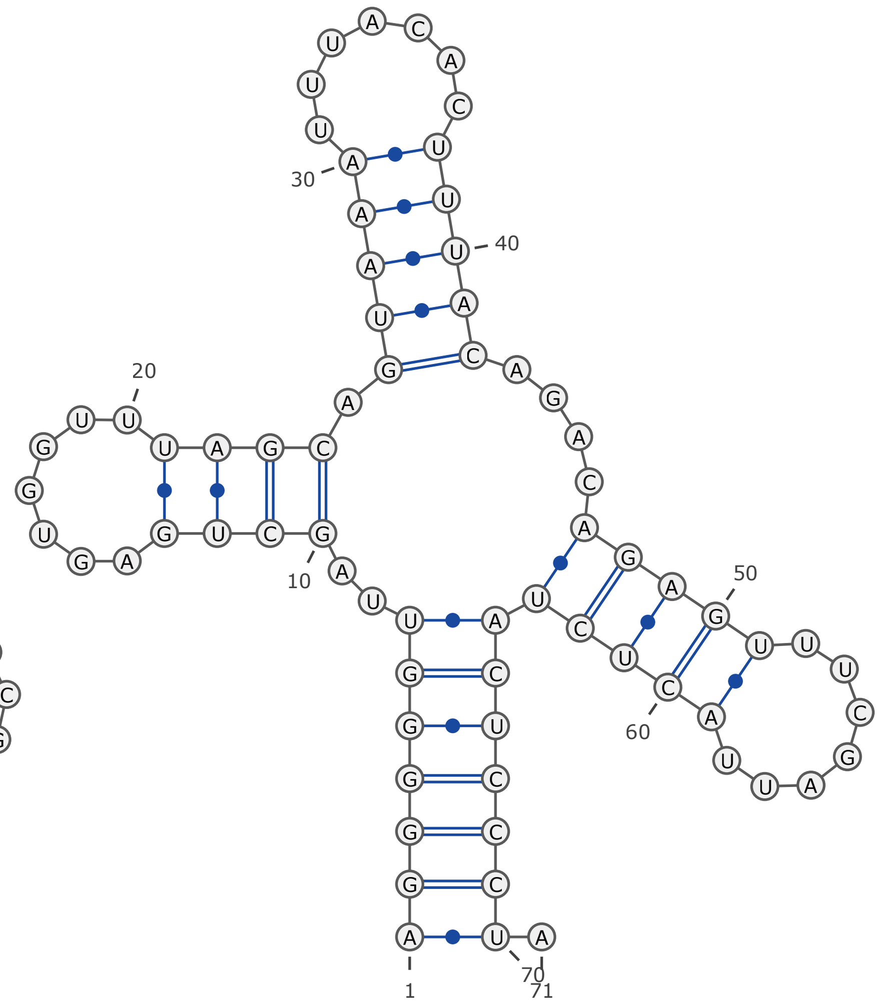

tRNA-VAL
