# Supplementary material for: Unveiling the Unique Mitogenome Structure of Phylloporus: Implications for Phylogeny and Evolution in Boletaceae
Source: J Fungi (Basel). 2025 Nov 25;11(12):831. doi: 10.3390/jof11120831 (PMC12733429; doi:10.3390/jof11120831)
Supplement: Supplementary file 1 [file jof-11-00831-s001.zip › FIG6-Structure prediction/Figure S4-Phylloporus rubrosquamosus.pdf]

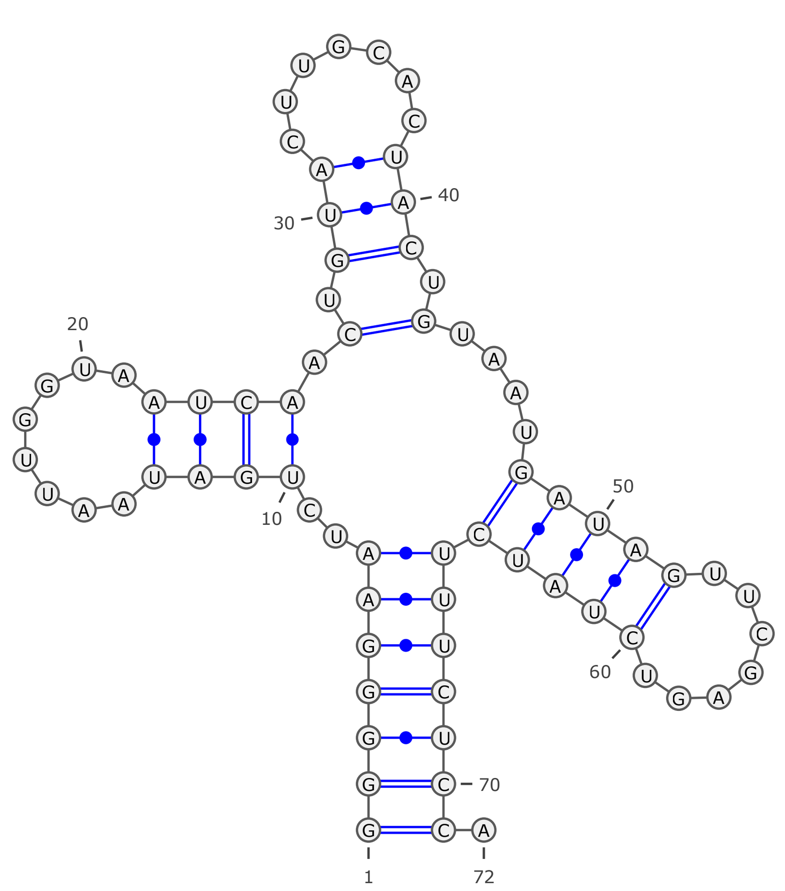
tRNA-ALA

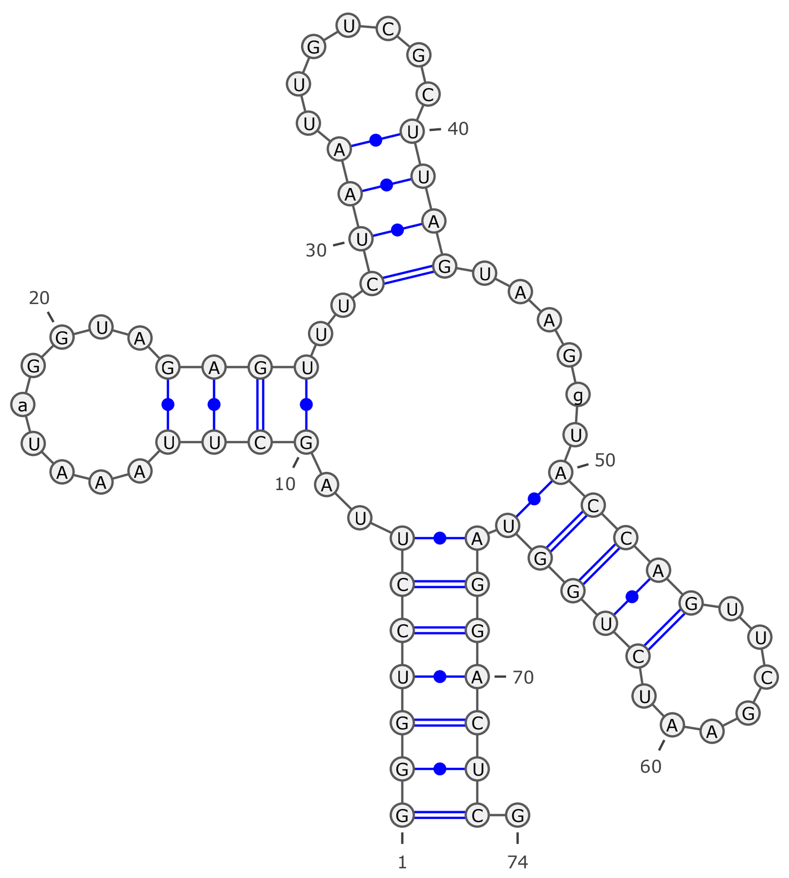
tRNA-ASP

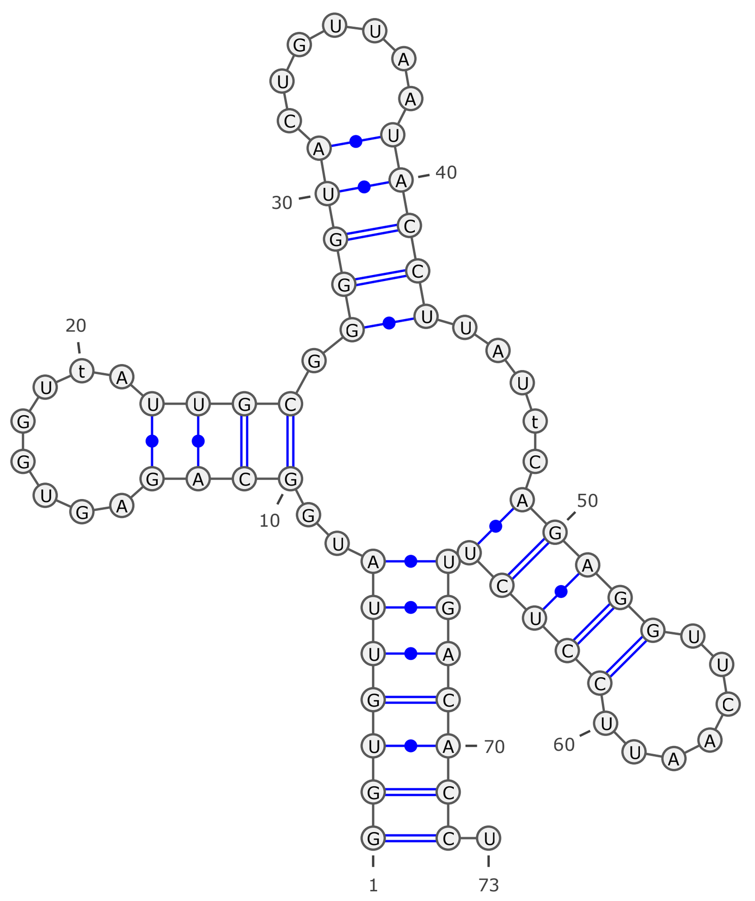
tRNA-ASN

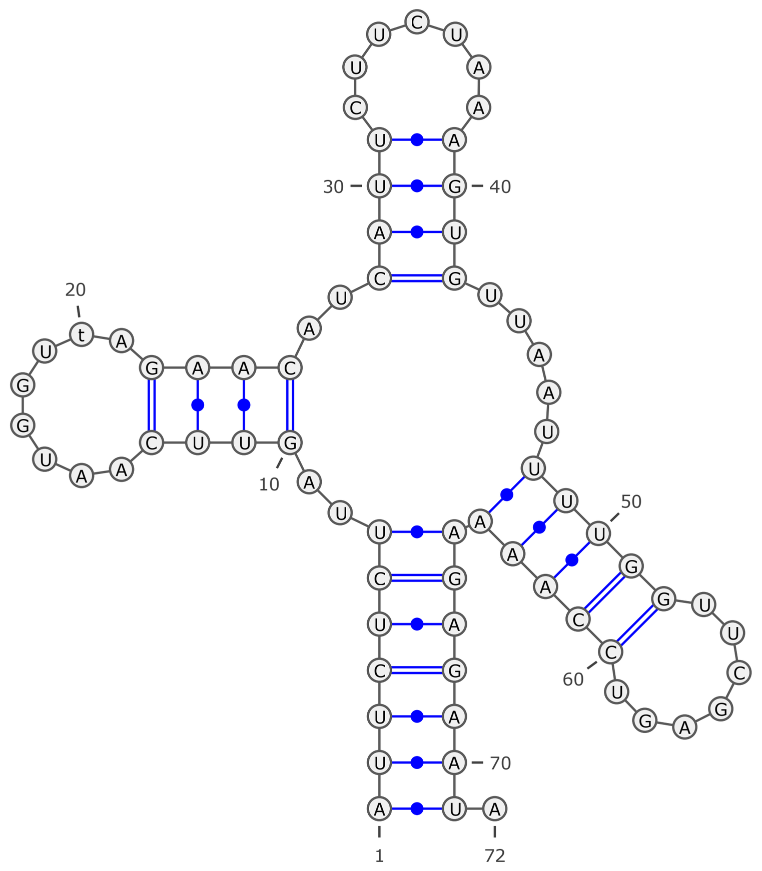
tRNA-ARG

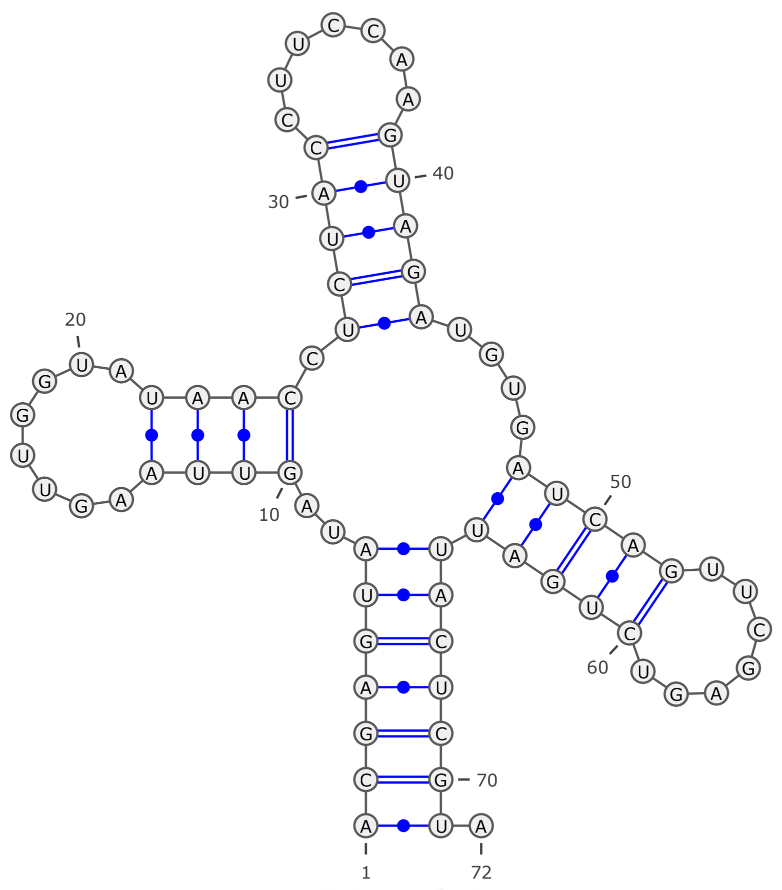
tRNA-GLY

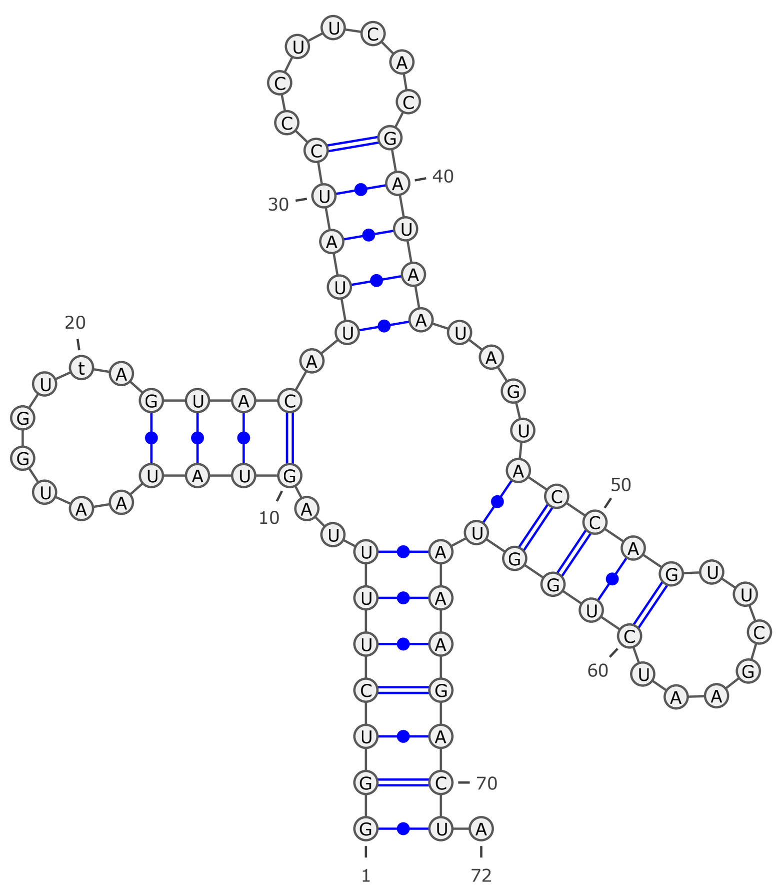
tRNA-GLU

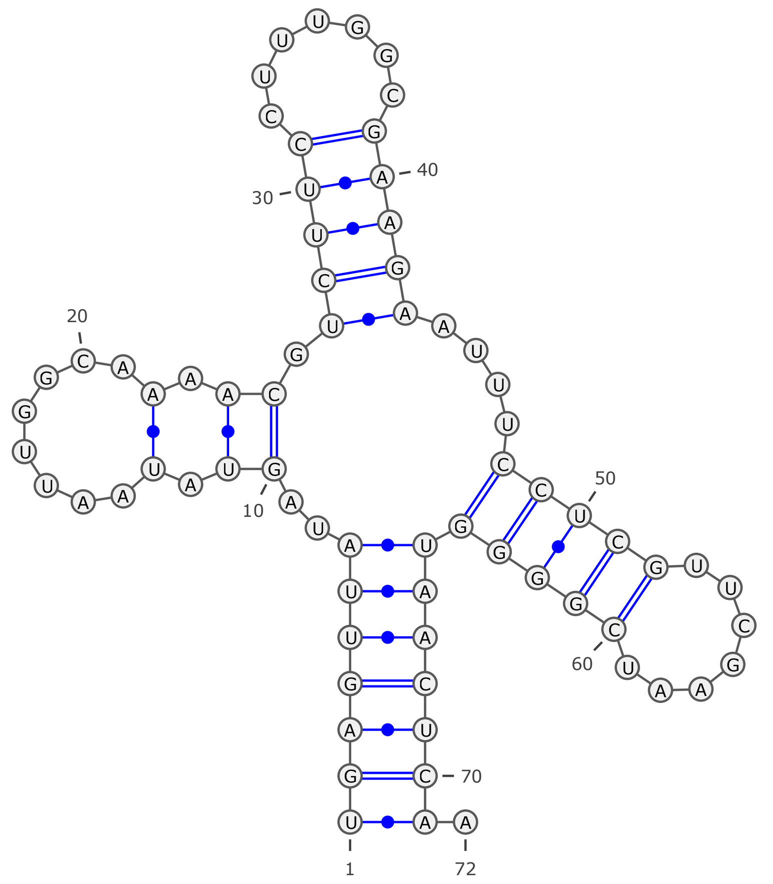
tRNA-GLN

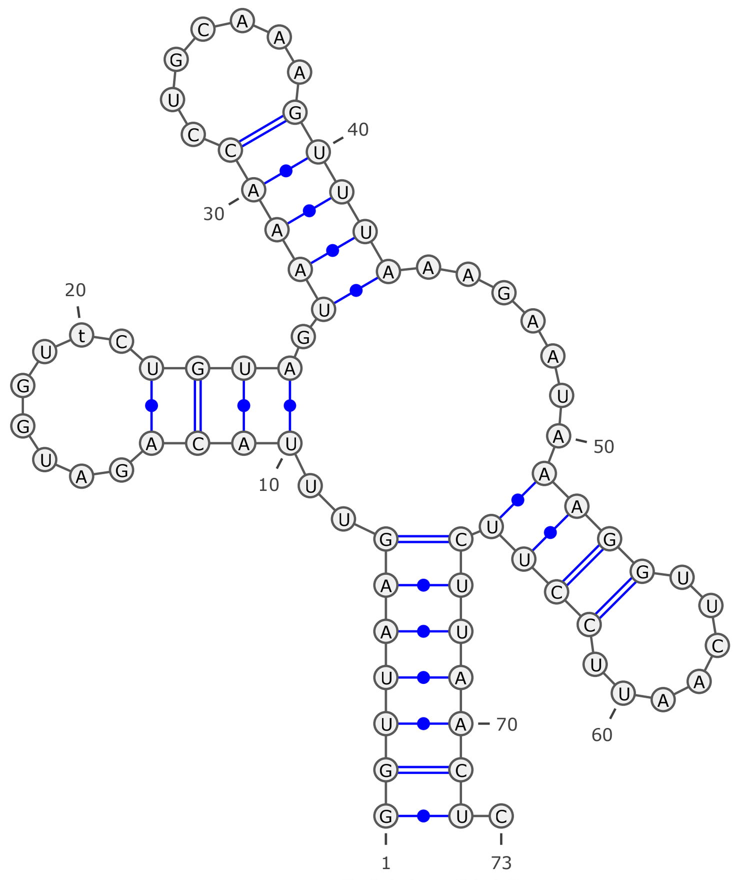
tRNA-CYS

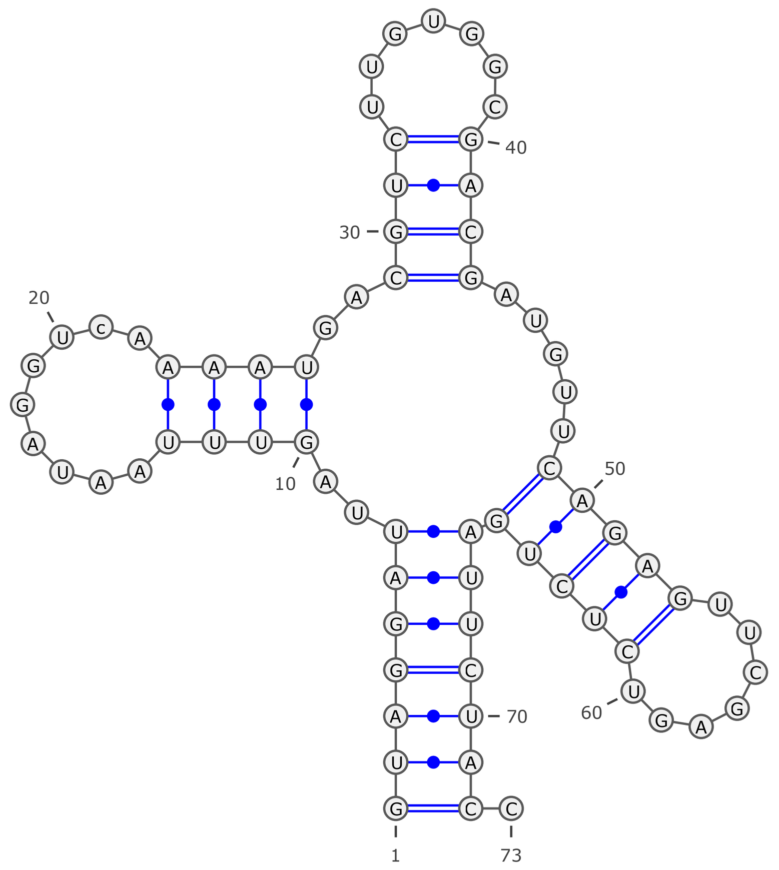
tRNA-HIS

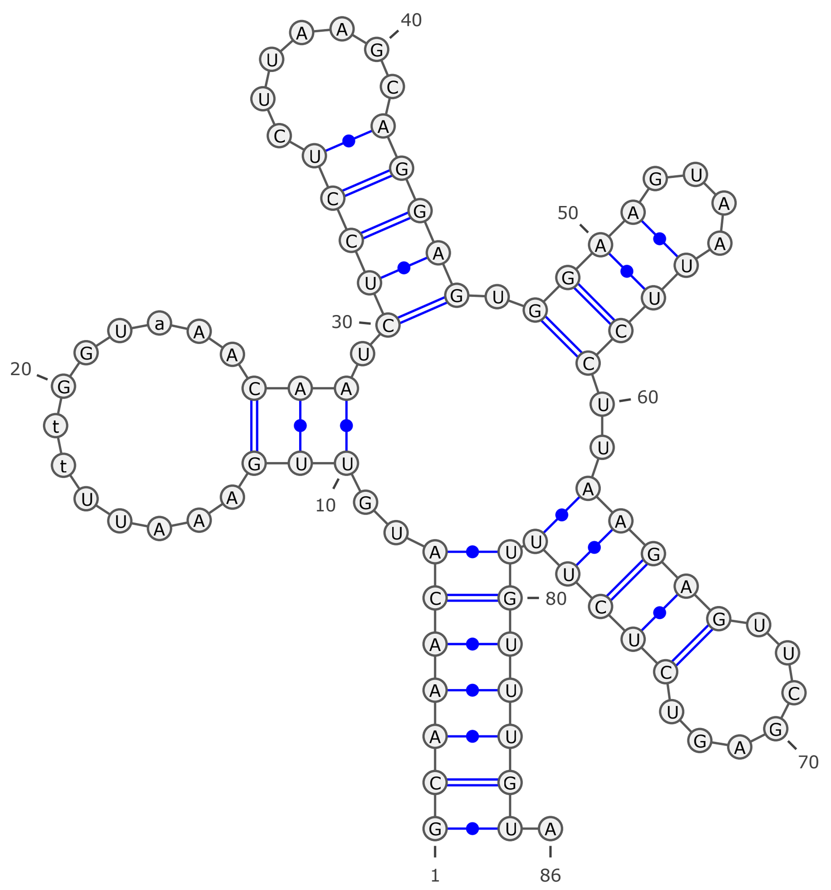
tRNA-LEU

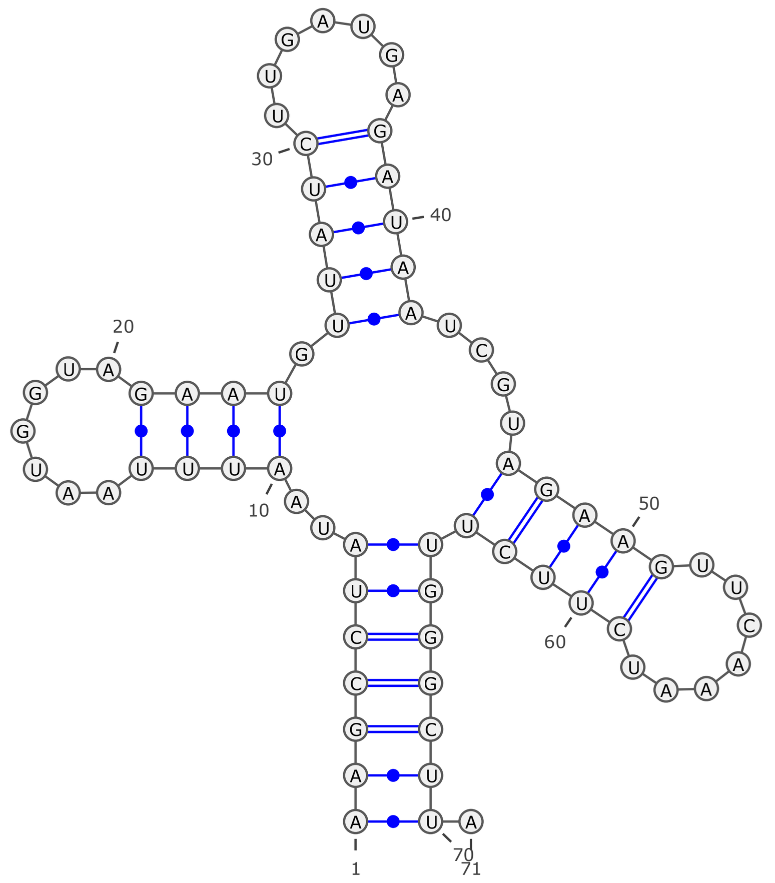
tRNA-LLE

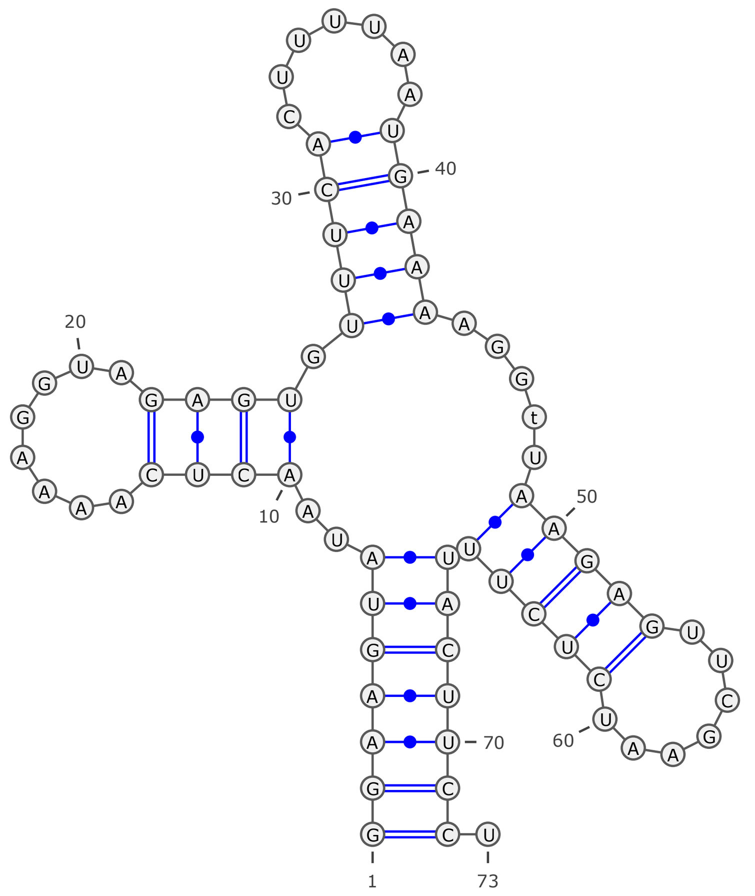
tRNA-LYS

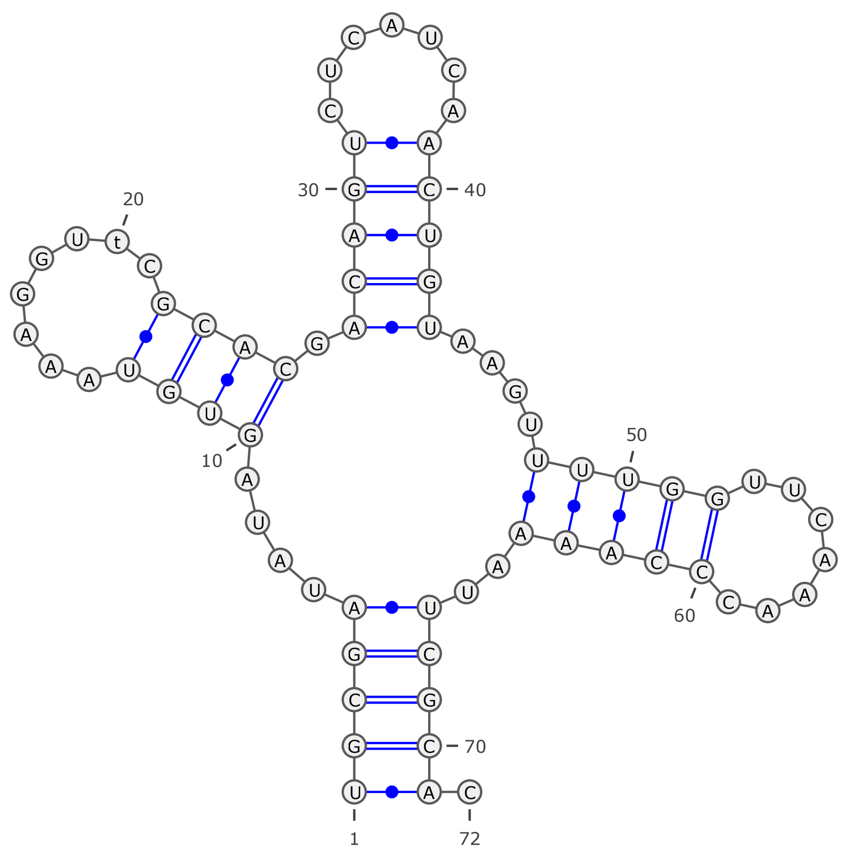
tRNA-MET

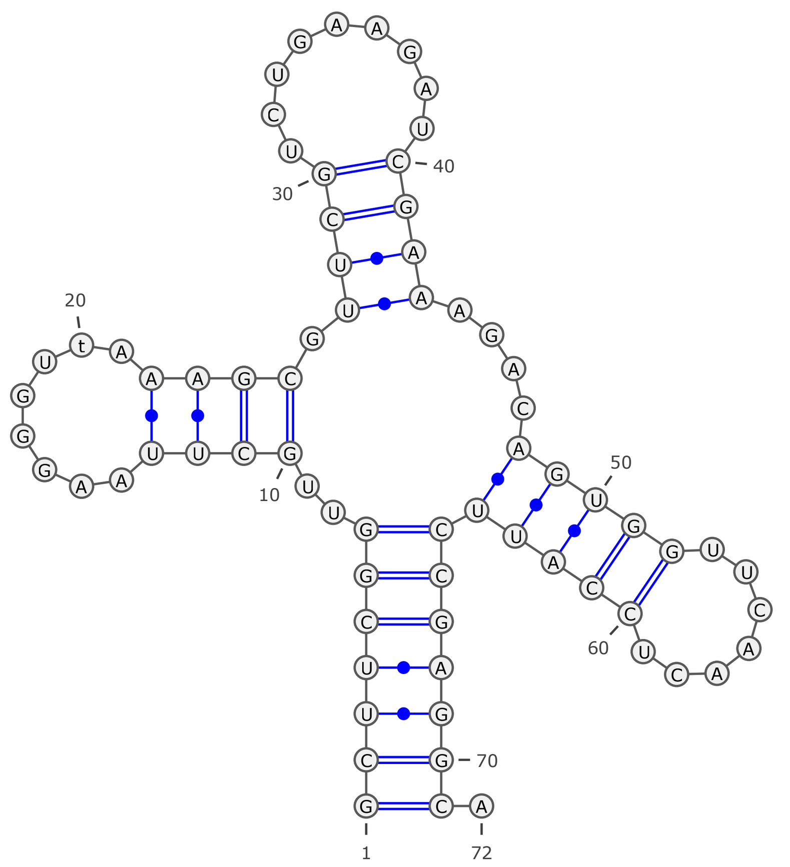
tRNA-PHE

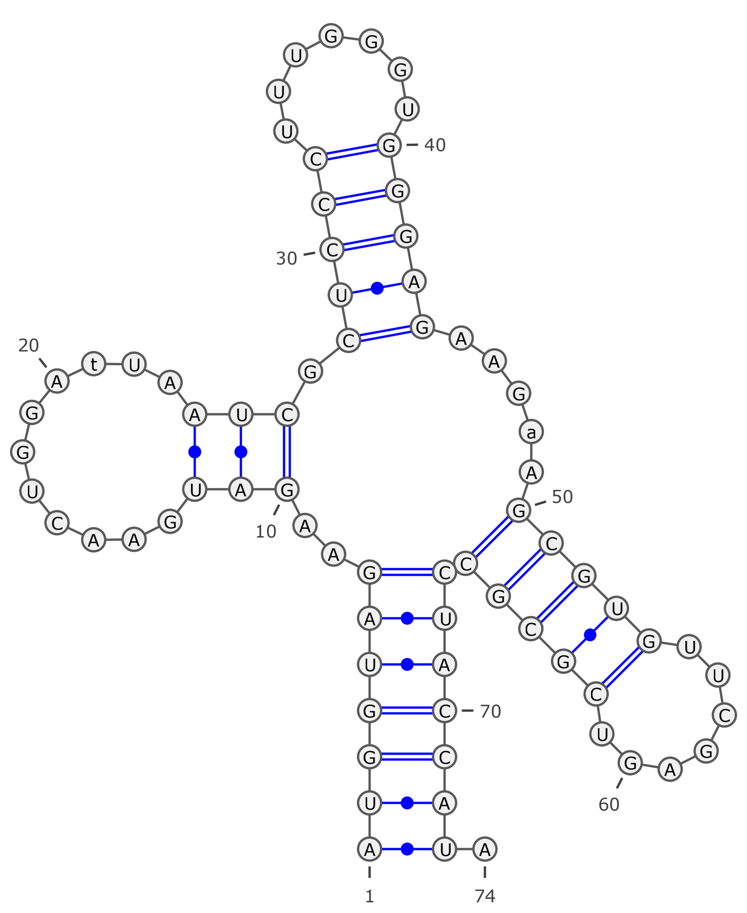
tRNA-PRO

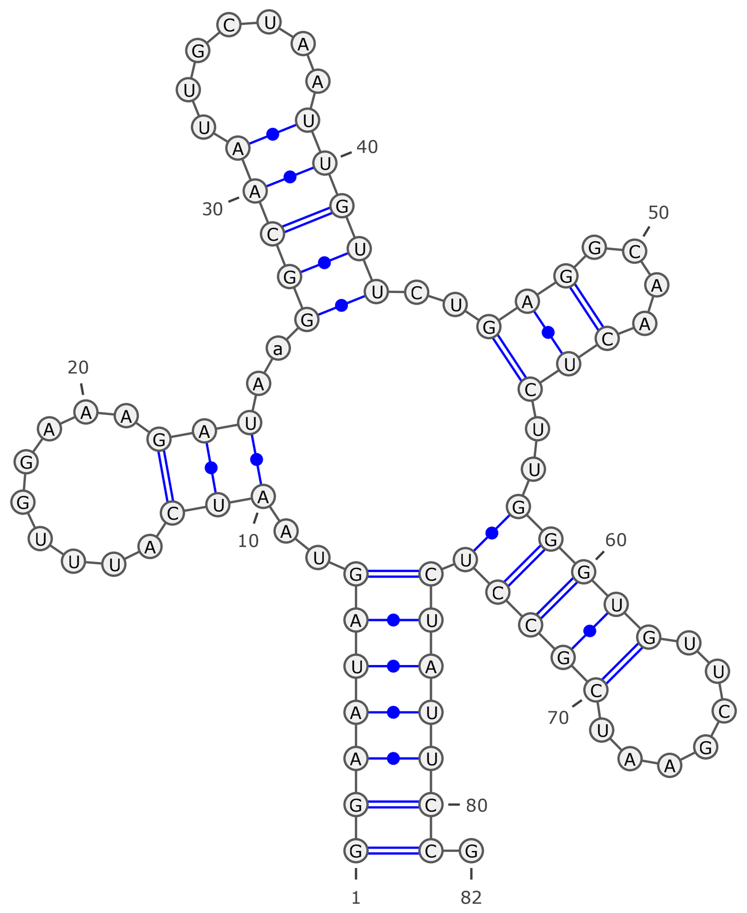
tRMA-SER

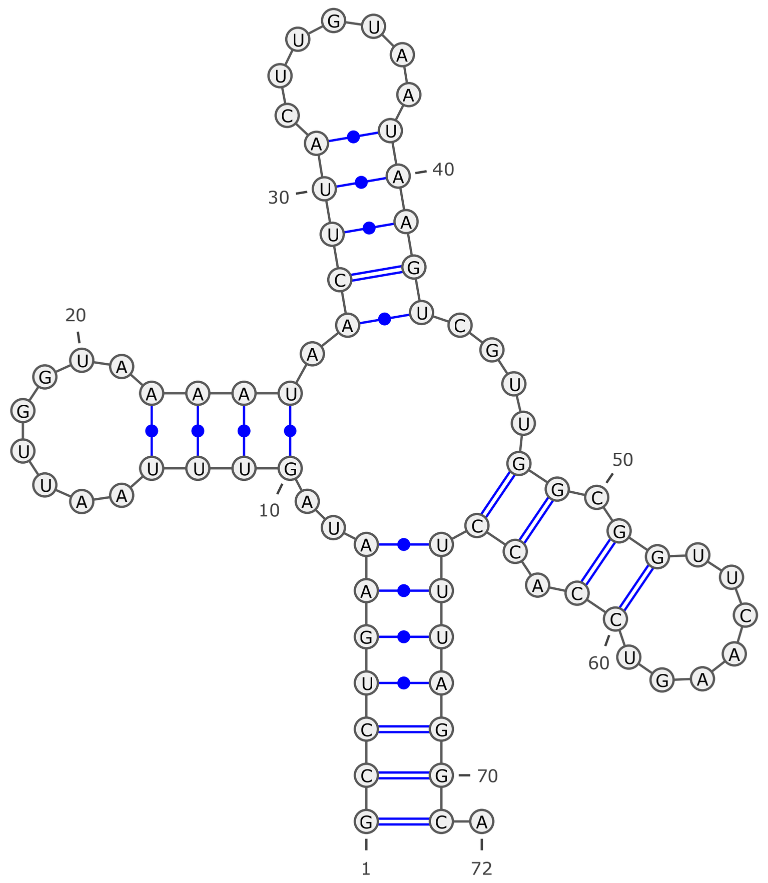
tRNA-THR

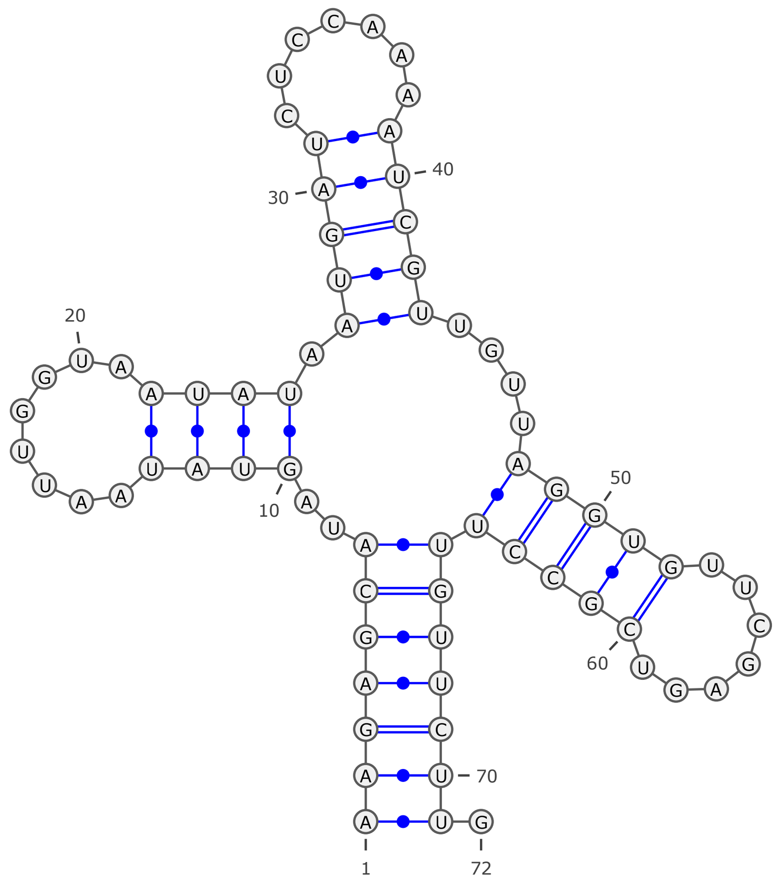
tRNA-TRP

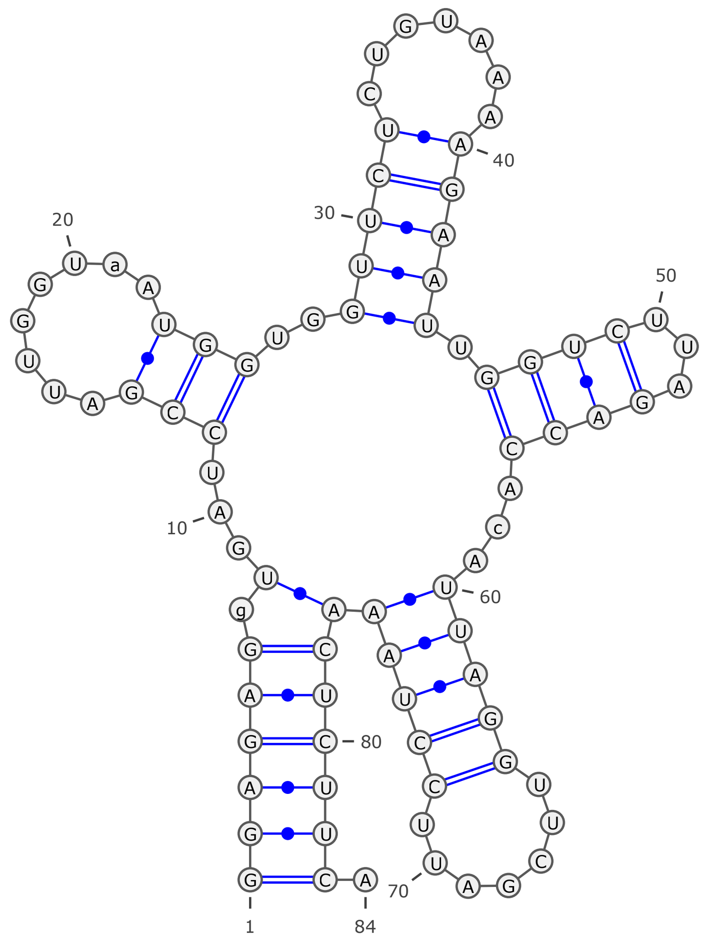
tRNA-TYR

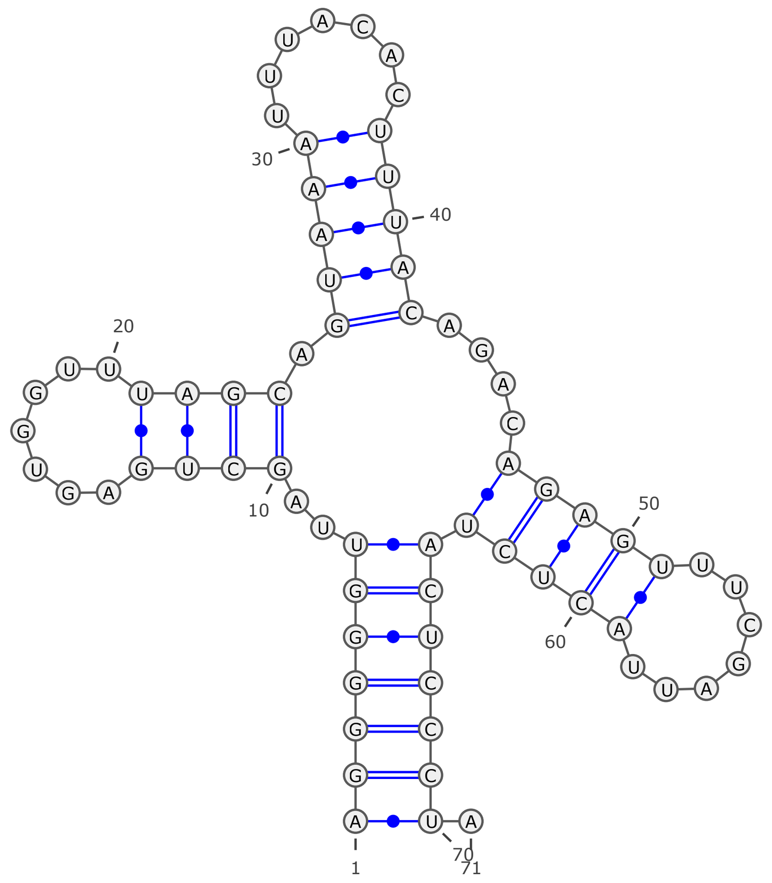
tRNA-VAL

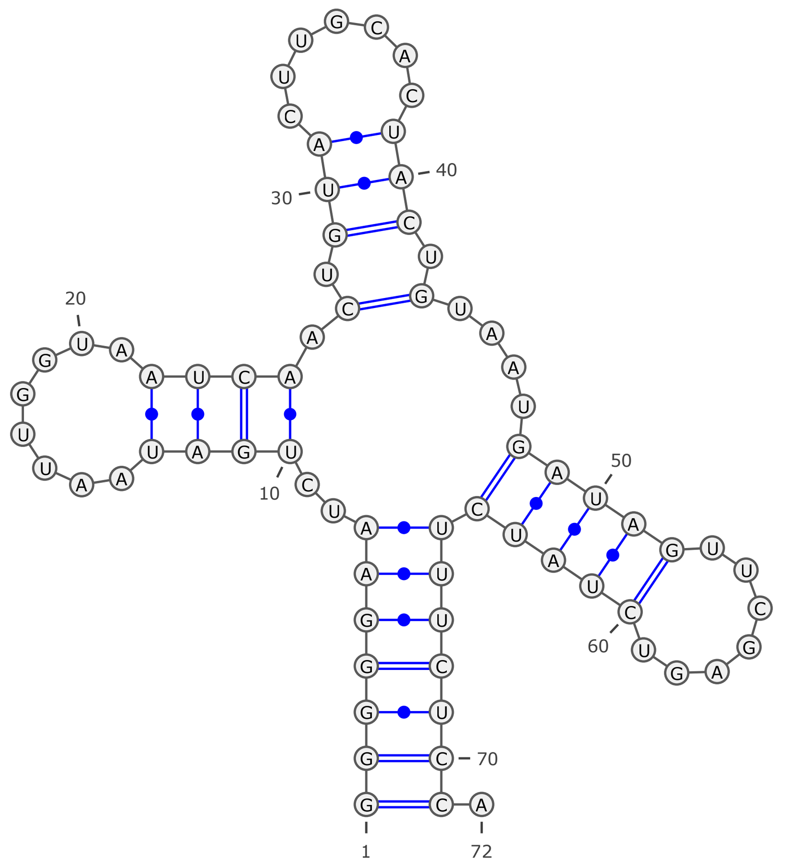
tRNA-ALA
