# Supplementary material for: Unveiling the Unique Mitogenome Structure of Phylloporus: Implications for Phylogeny and Evolution in Boletaceae
Source: J Fungi (Basel). 2025 Nov 25;11(12):831. doi: 10.3390/jof11120831 (PMC12733429; doi:10.3390/jof11120831)
Supplement: Supplementary file 1 [file jof-11-00831-s001.zip › FIG6-Structure prediction/Figure S6-Phylloprus grossus1301.pdf]

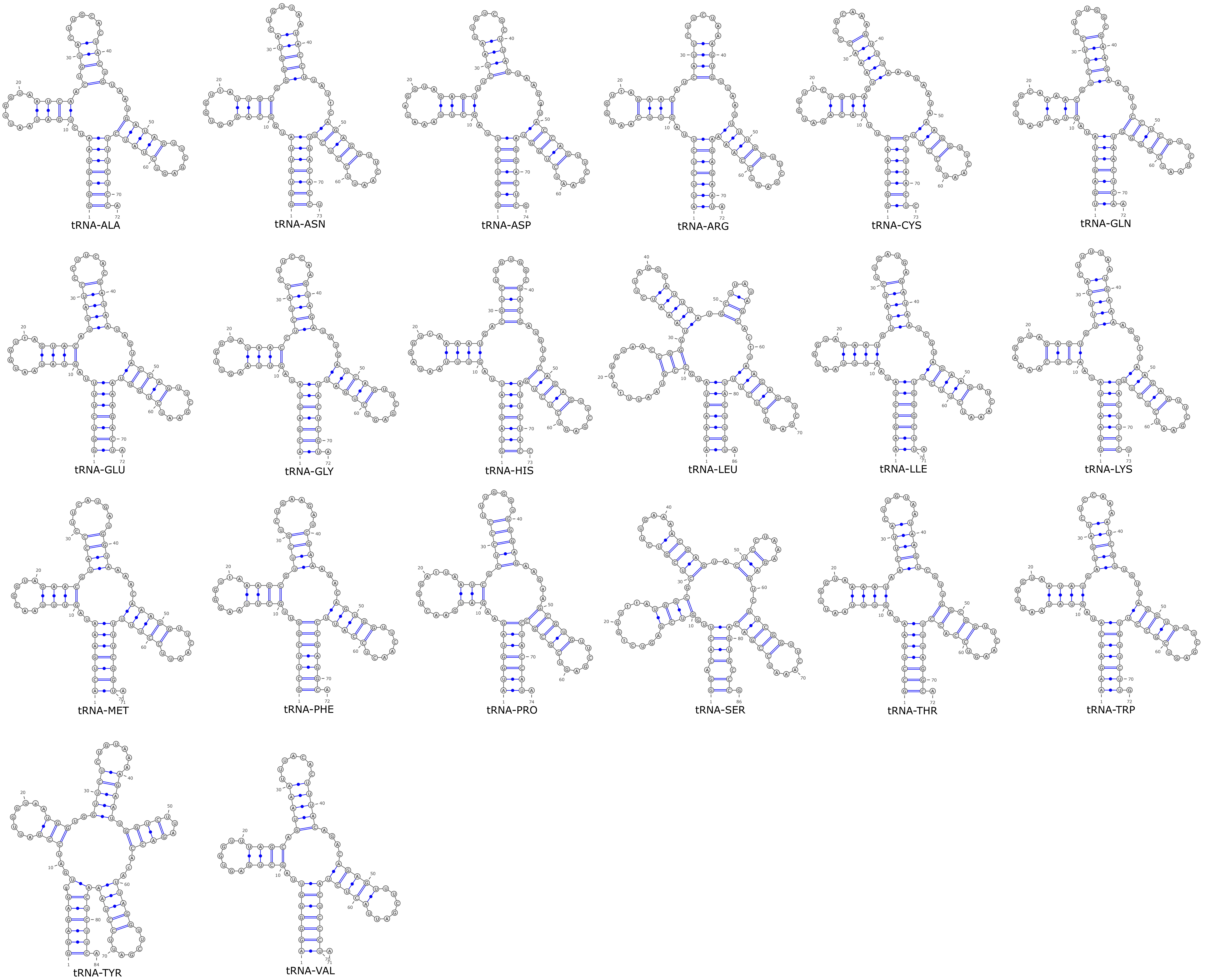
tRNA-ALA
tRNA-ASN
tRNA-ASP
tRNA-ARG
tRNA-CYS
tRNA-GLN
tRNA-GLU
tRNA-GLY
tRNA-HIS
tRNA-LEU
tRNA-LLE
tRNA-LYS
tRNA-MET
tRNA-PHE
tRNA-PRO
tRNA-SER
tRNA-THR
tRNA-TRP
tRNA-TYR
tRNA-VAL
