# Supplementary figures and images for: Unveiling the Unique Mitogenome Structure of Phylloporus: Implications for Phylogeny and Evolution in Boletaceae
Source: J Fungi (Basel). 2025 Nov 25;11(12):831. doi: 10.3390/jof11120831 (PMC12733429; doi:10.3390/jof11120831)

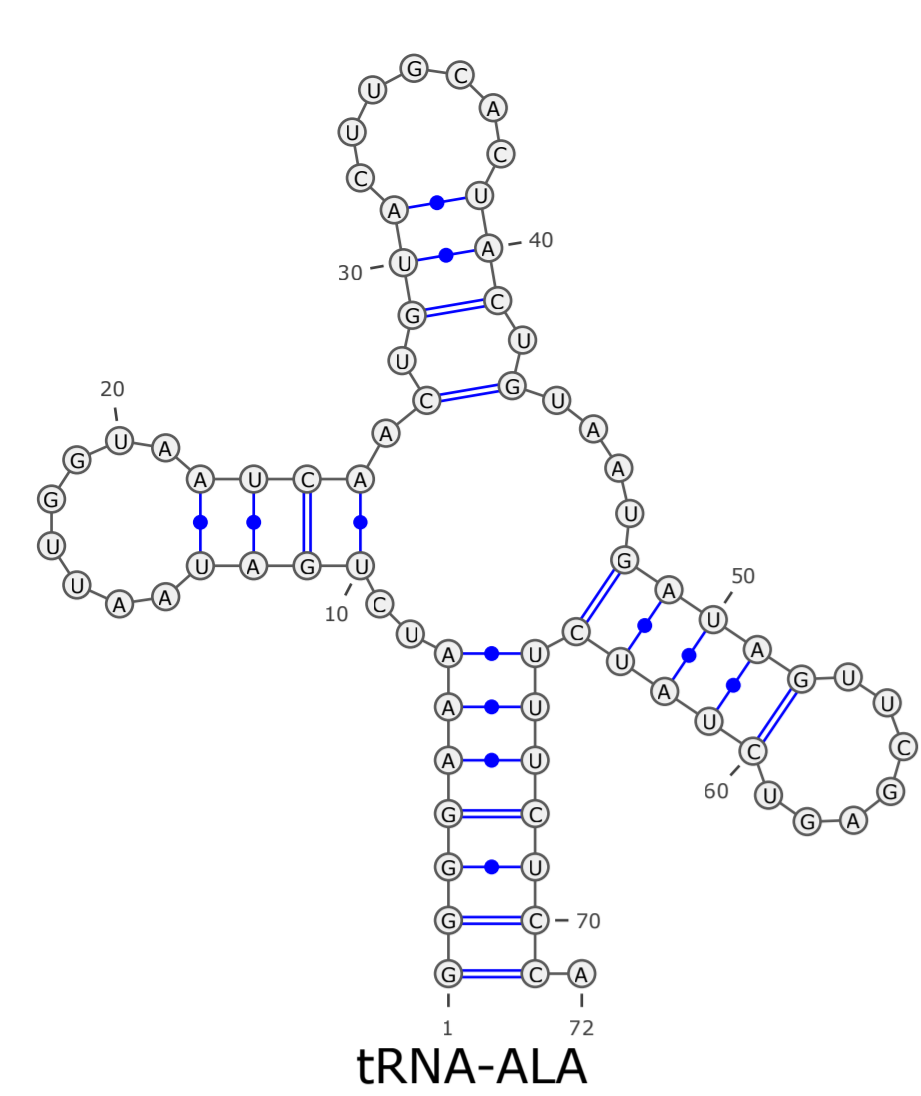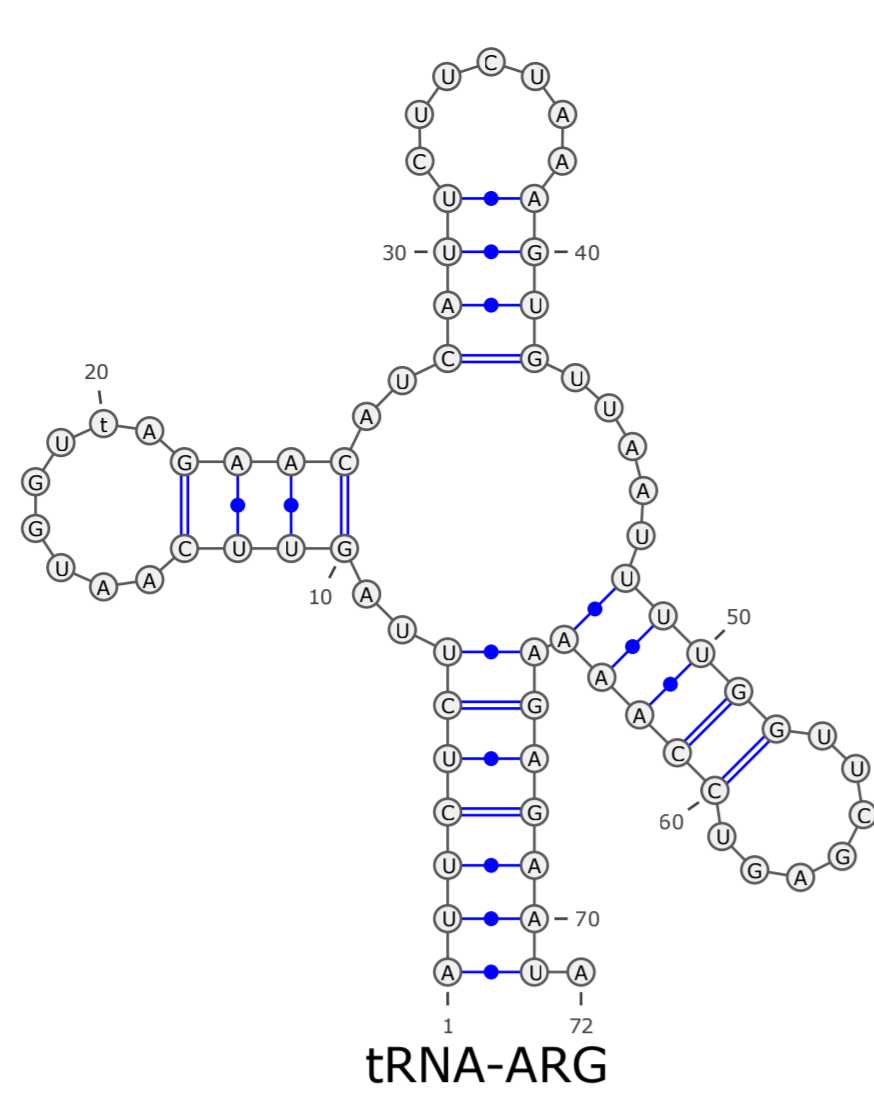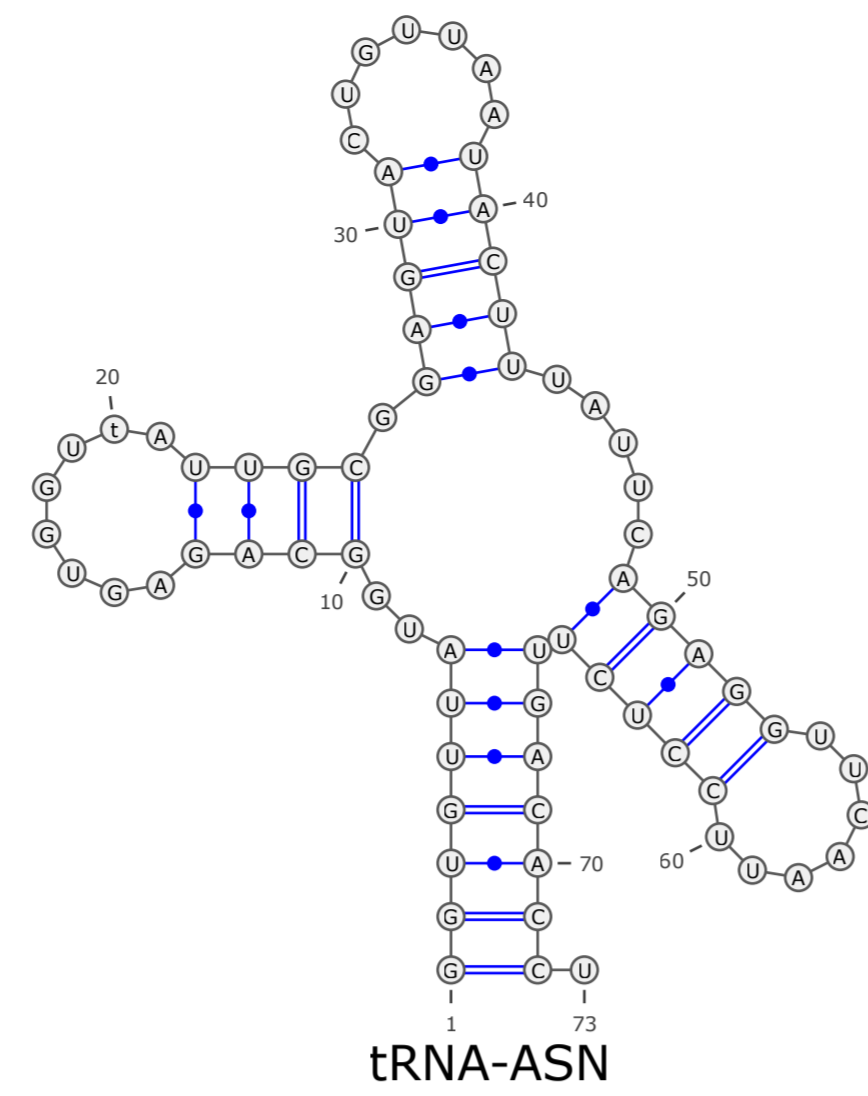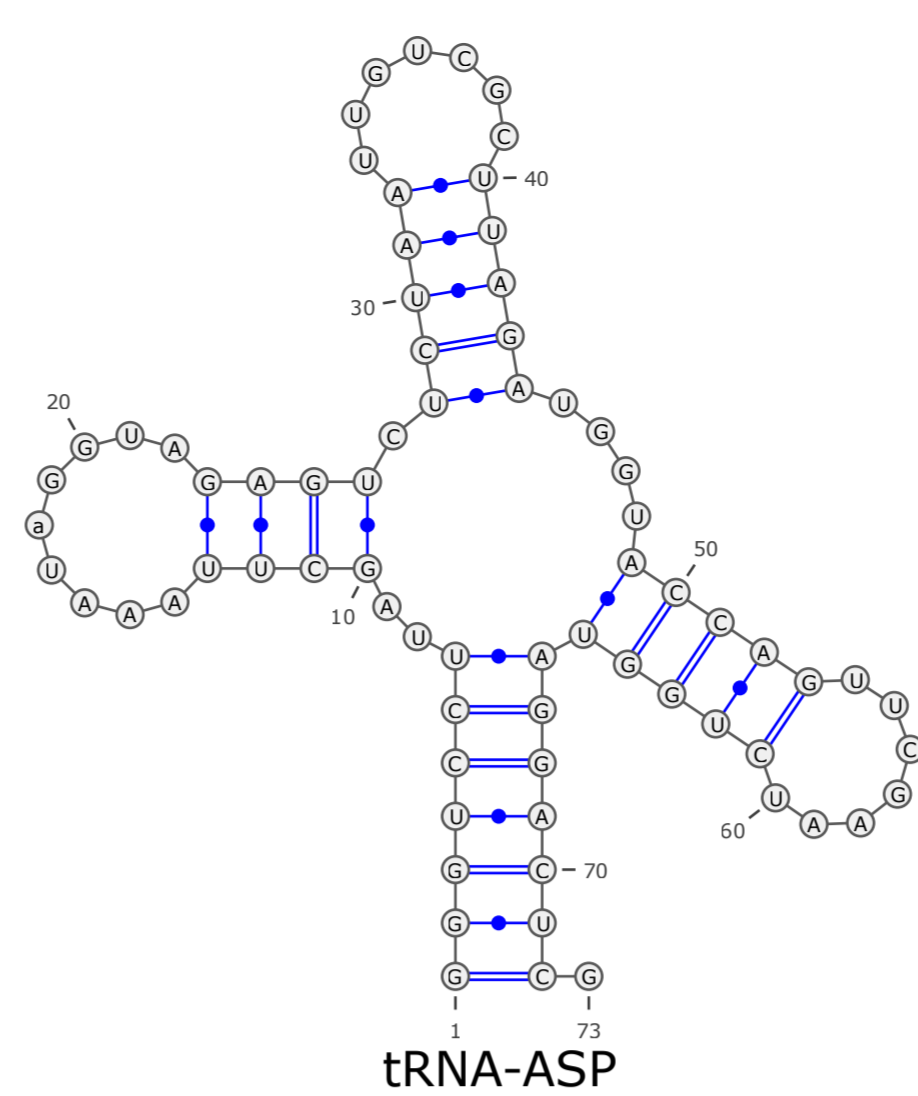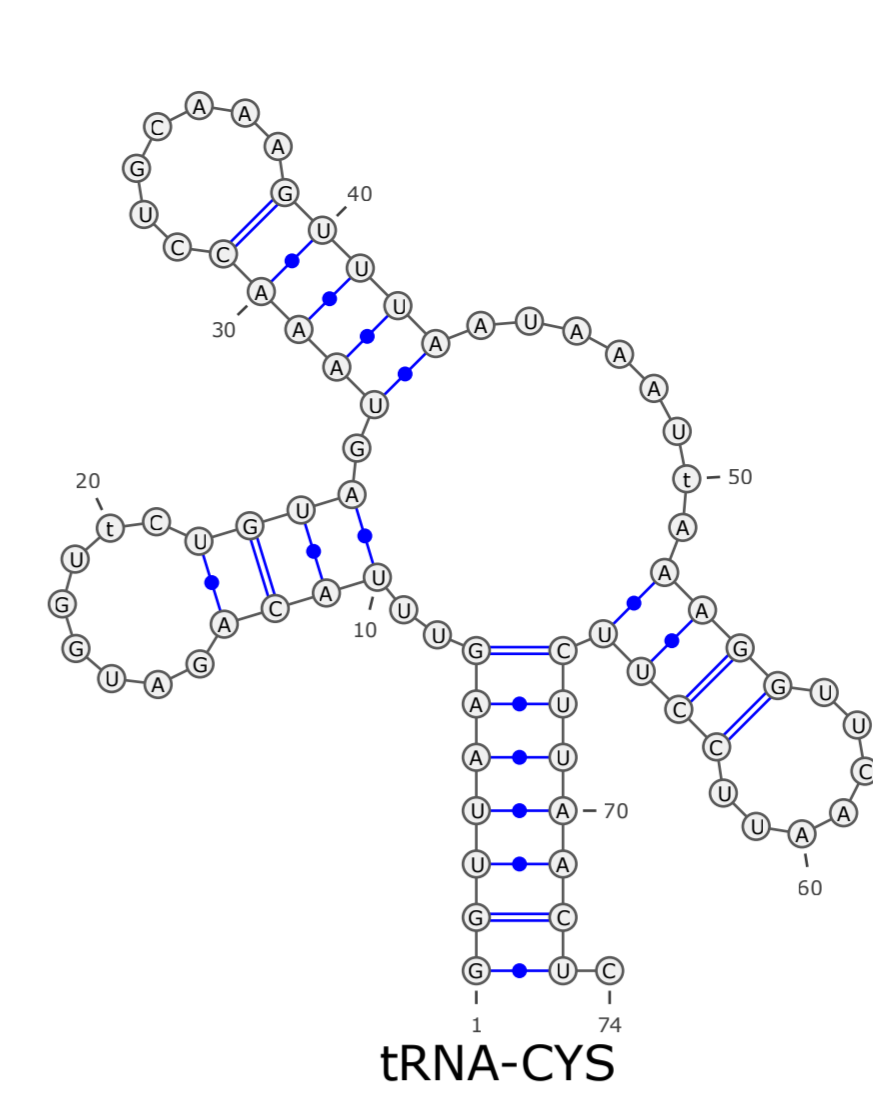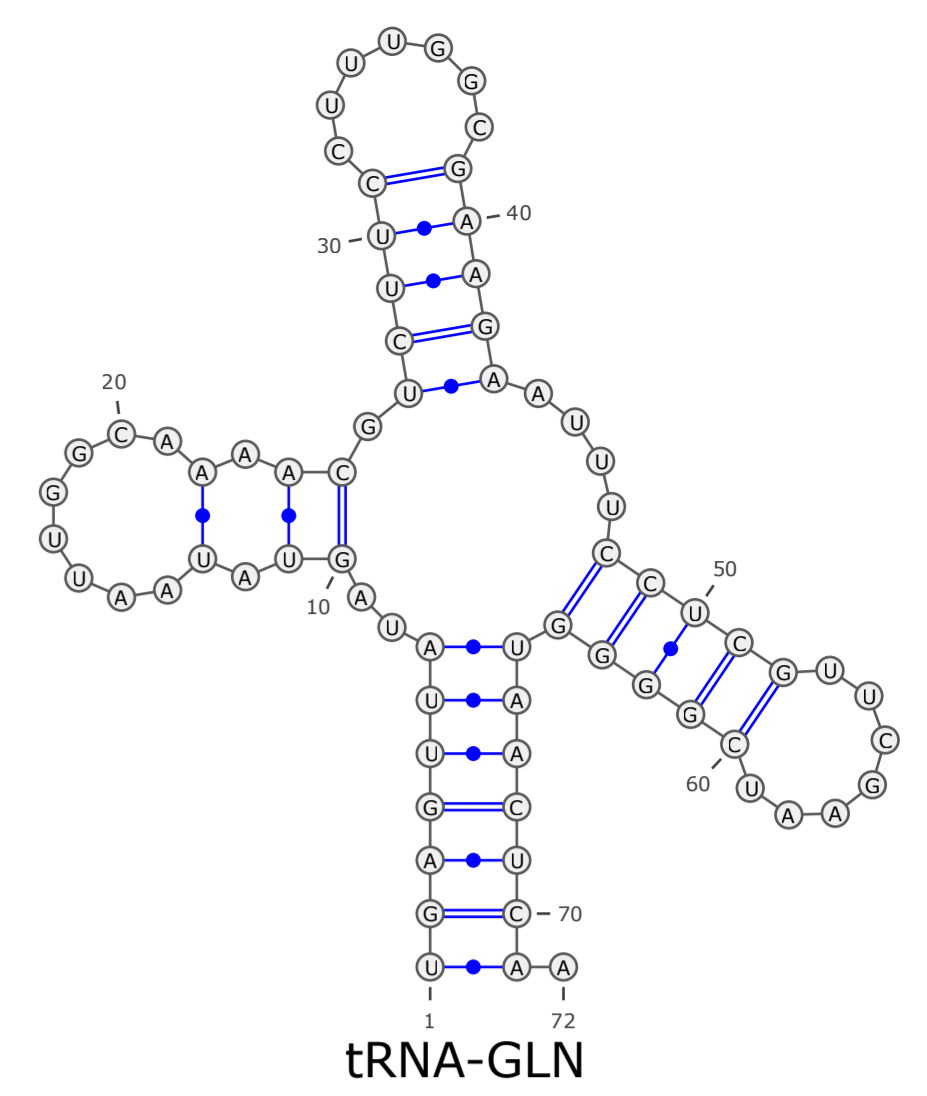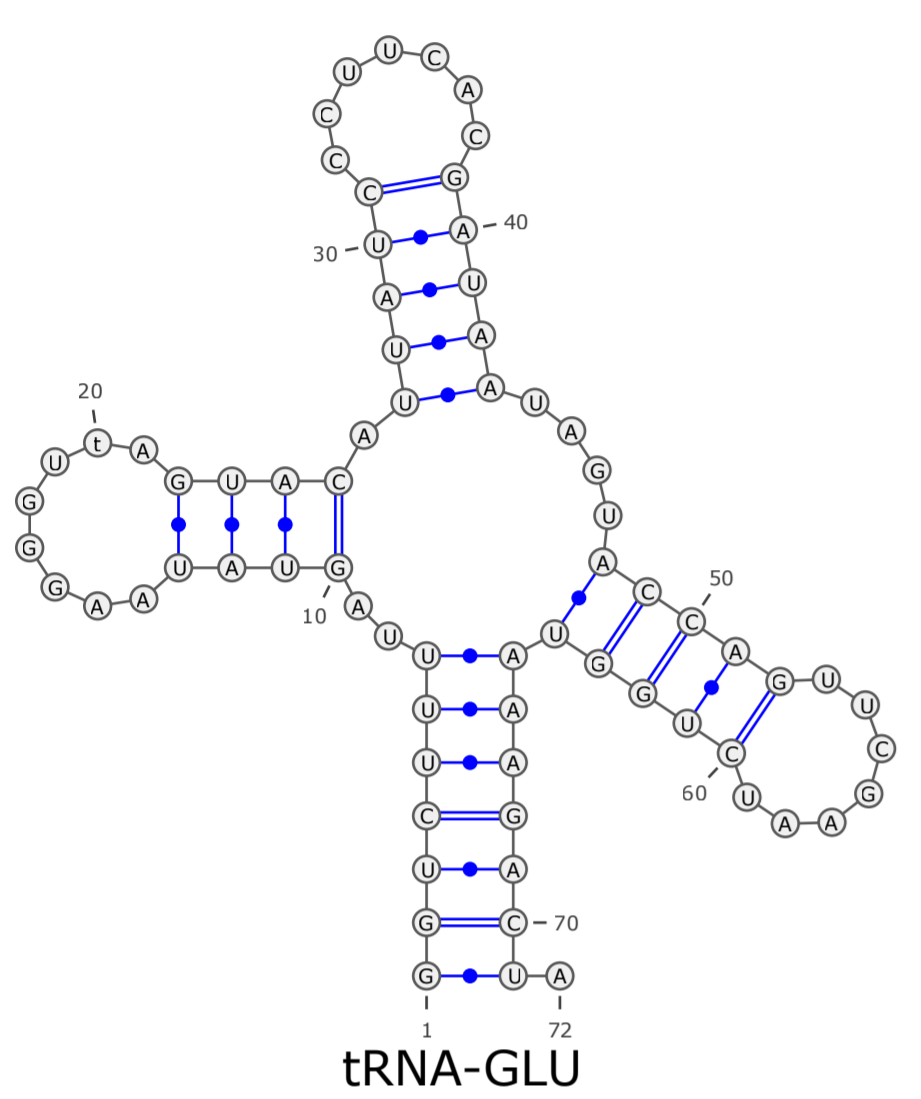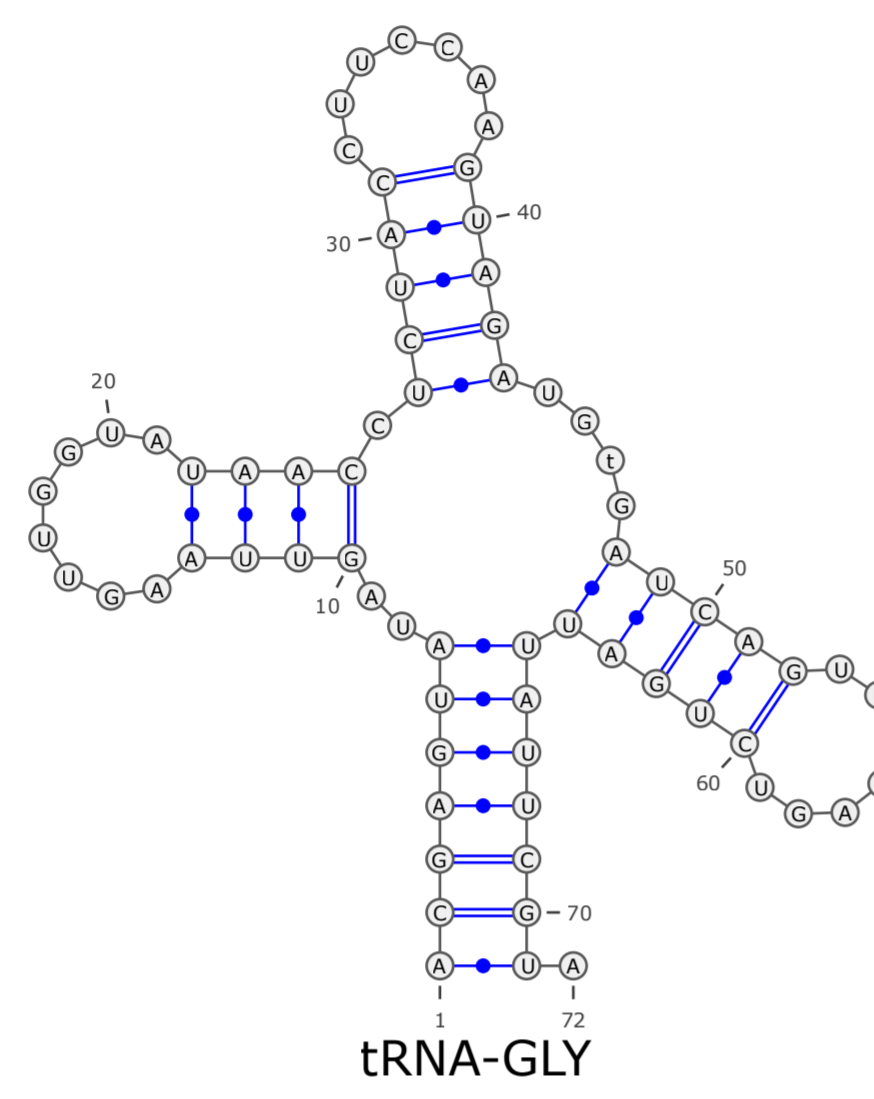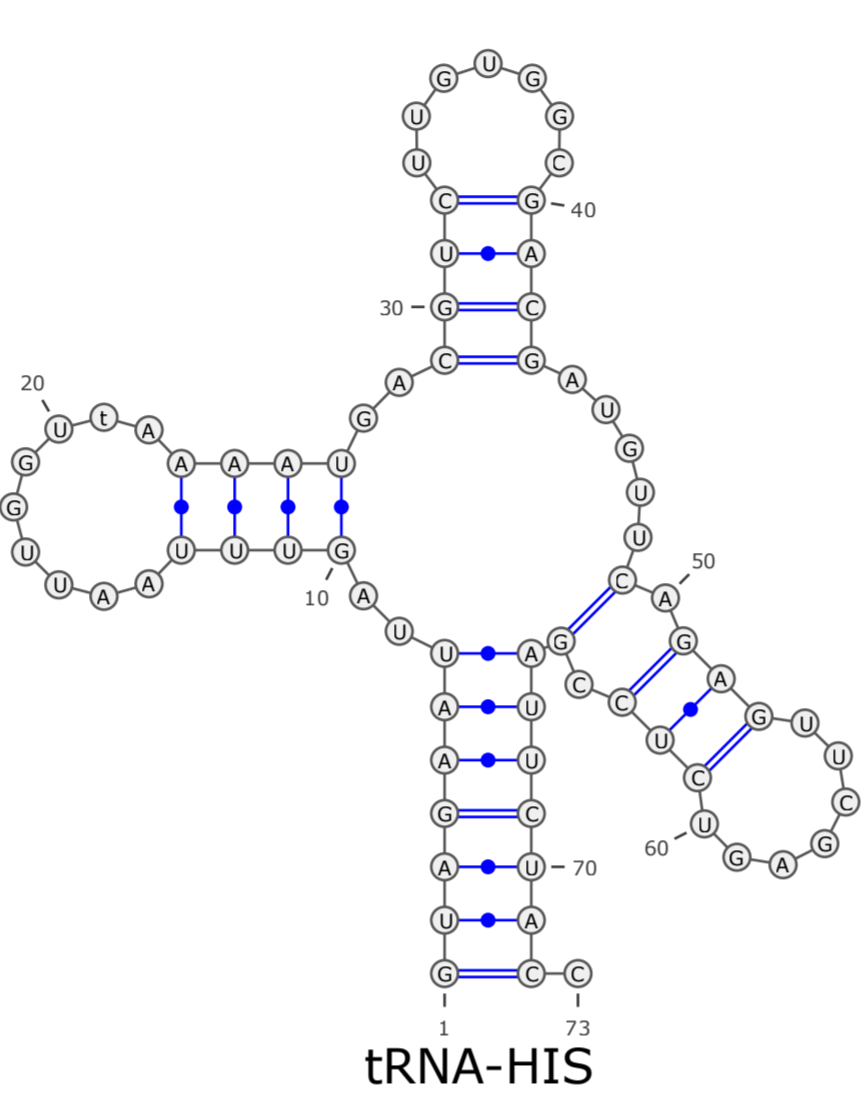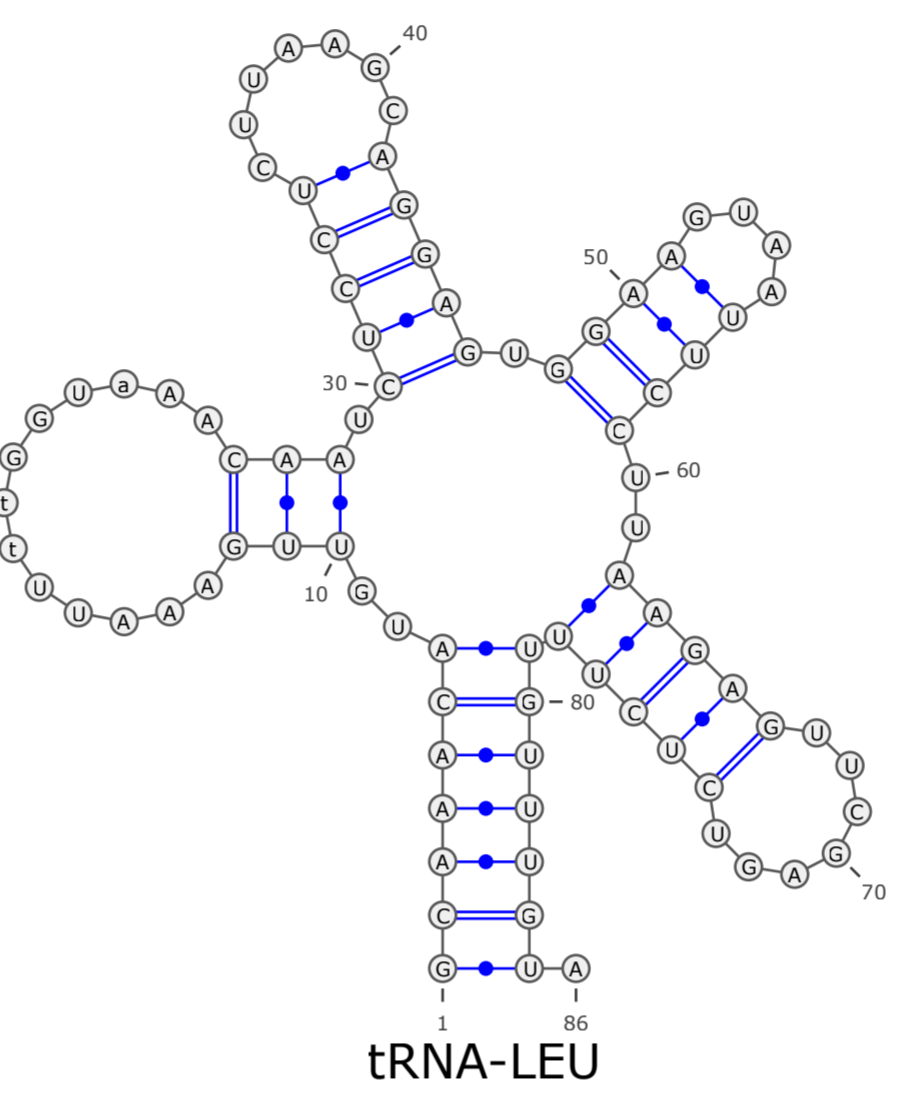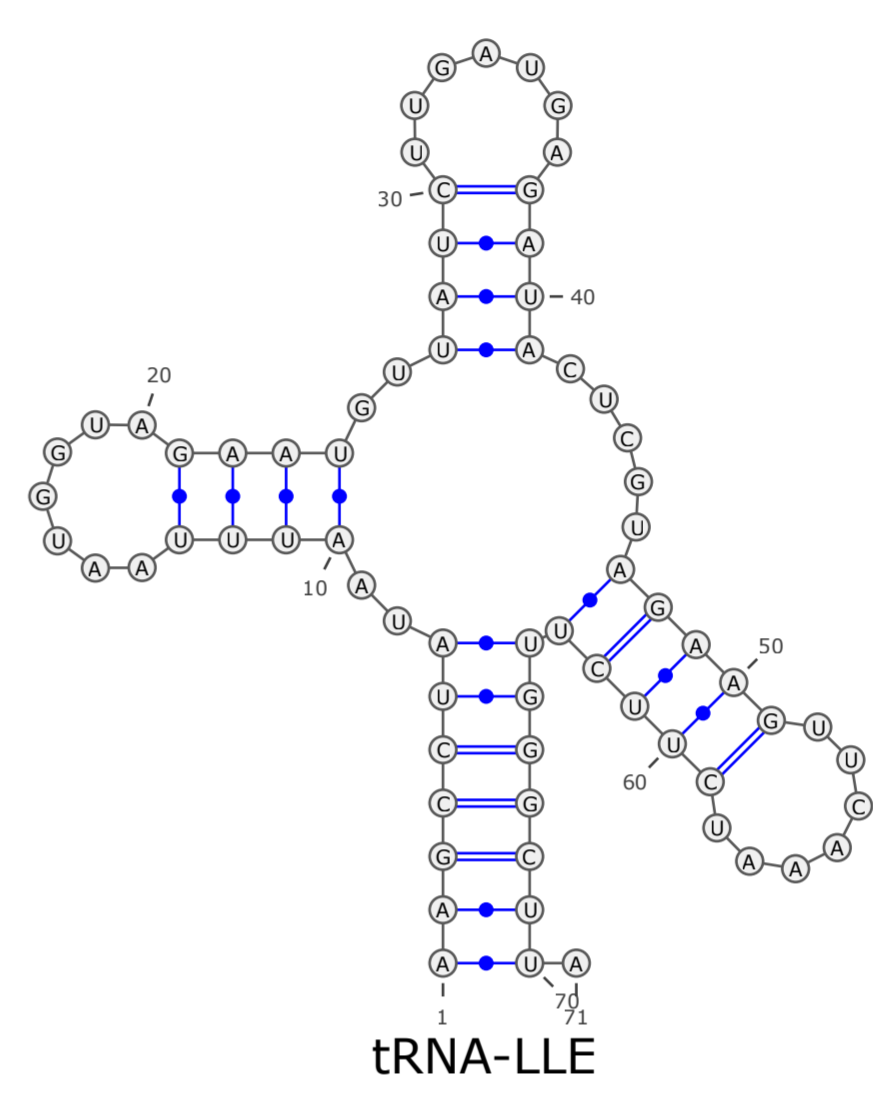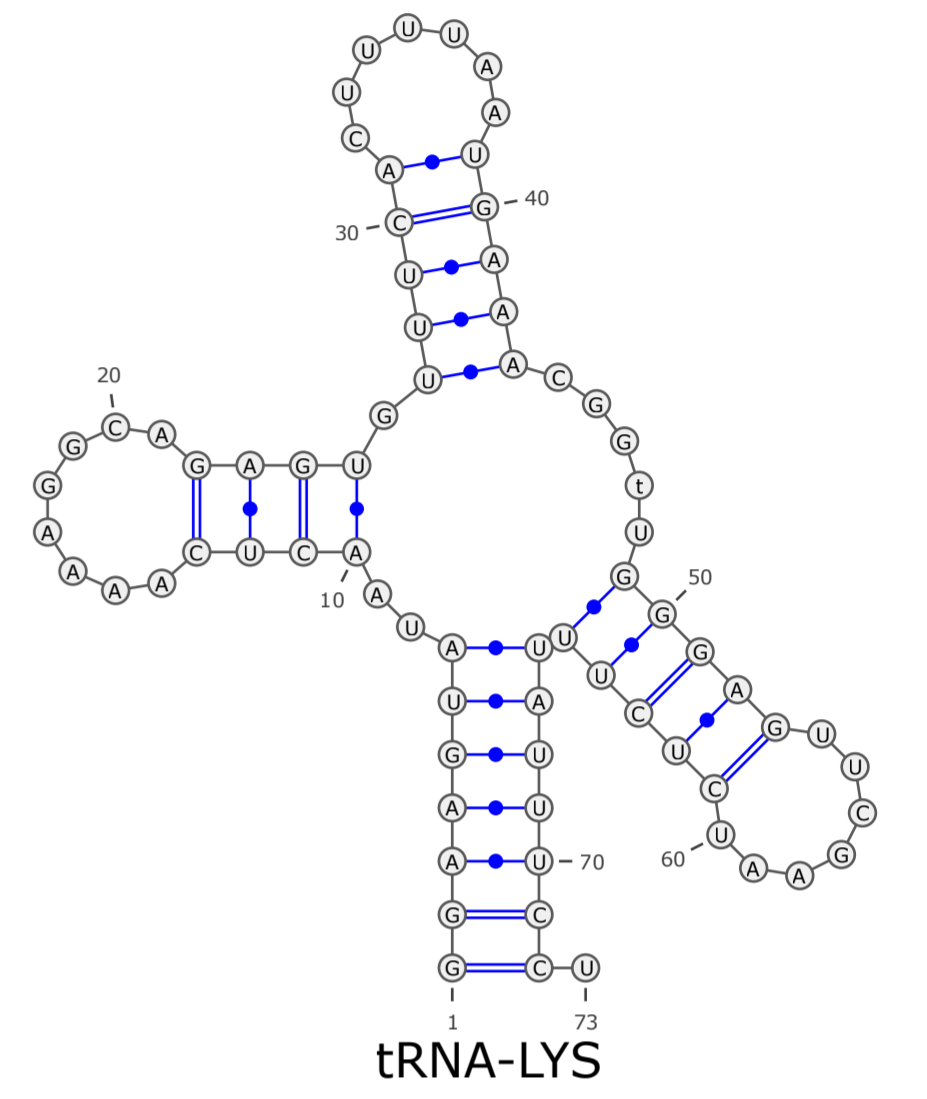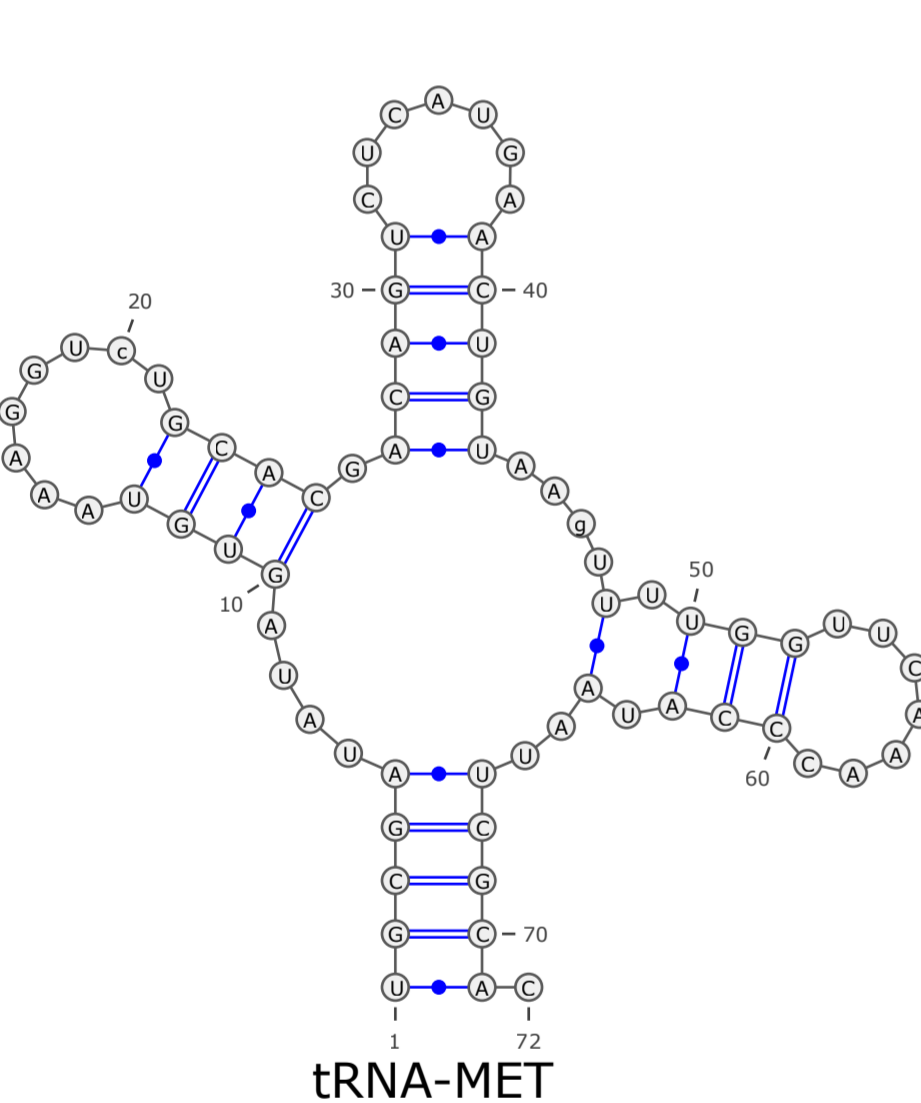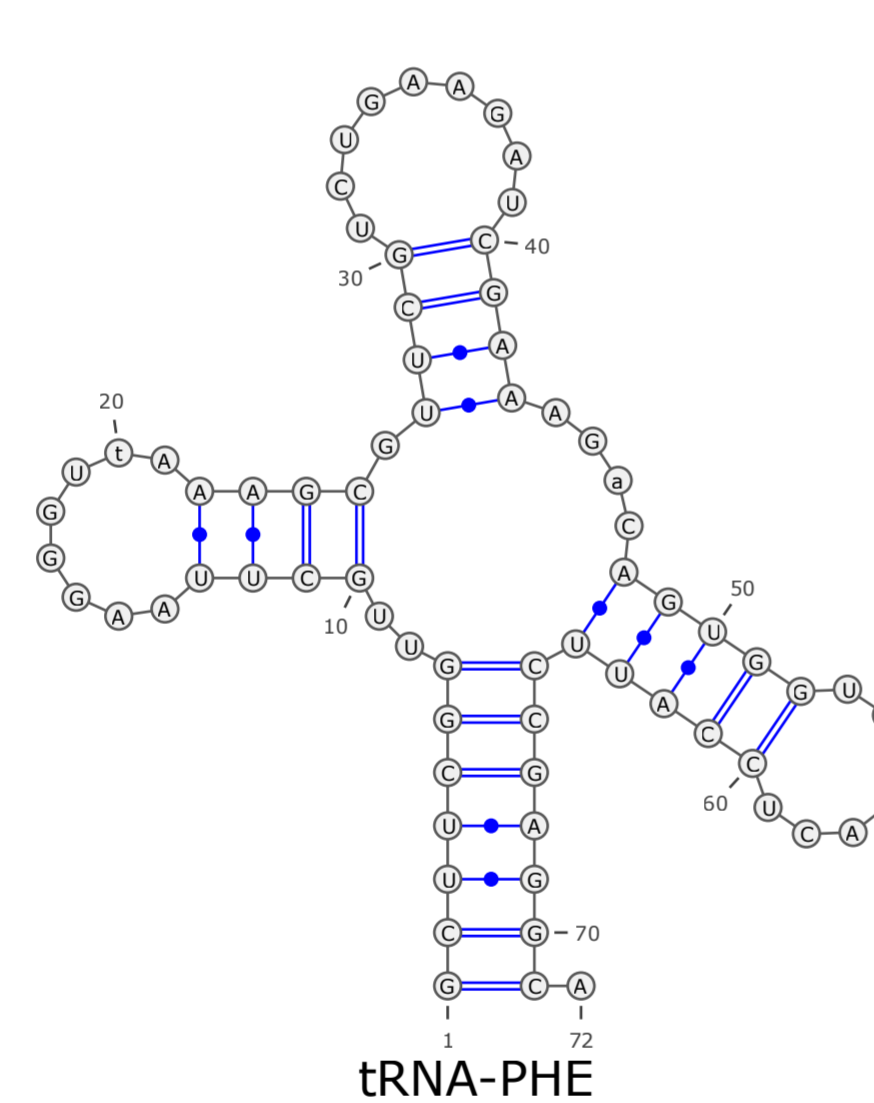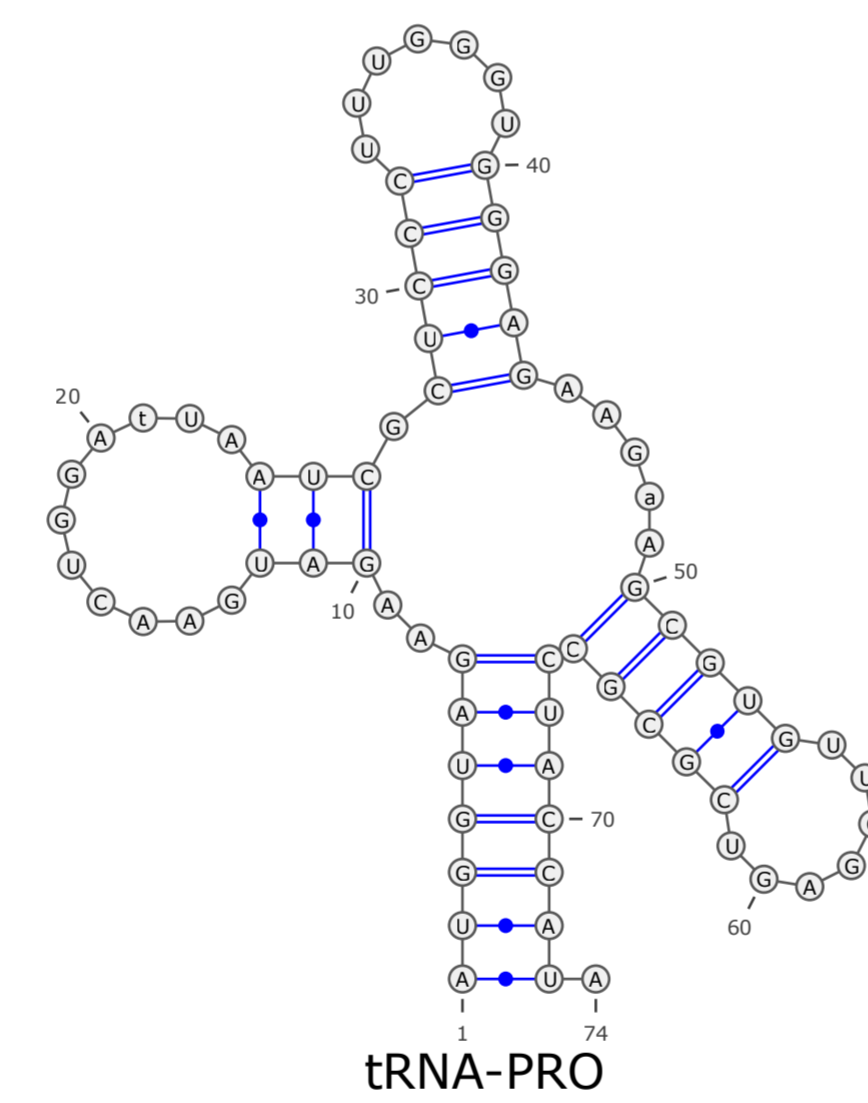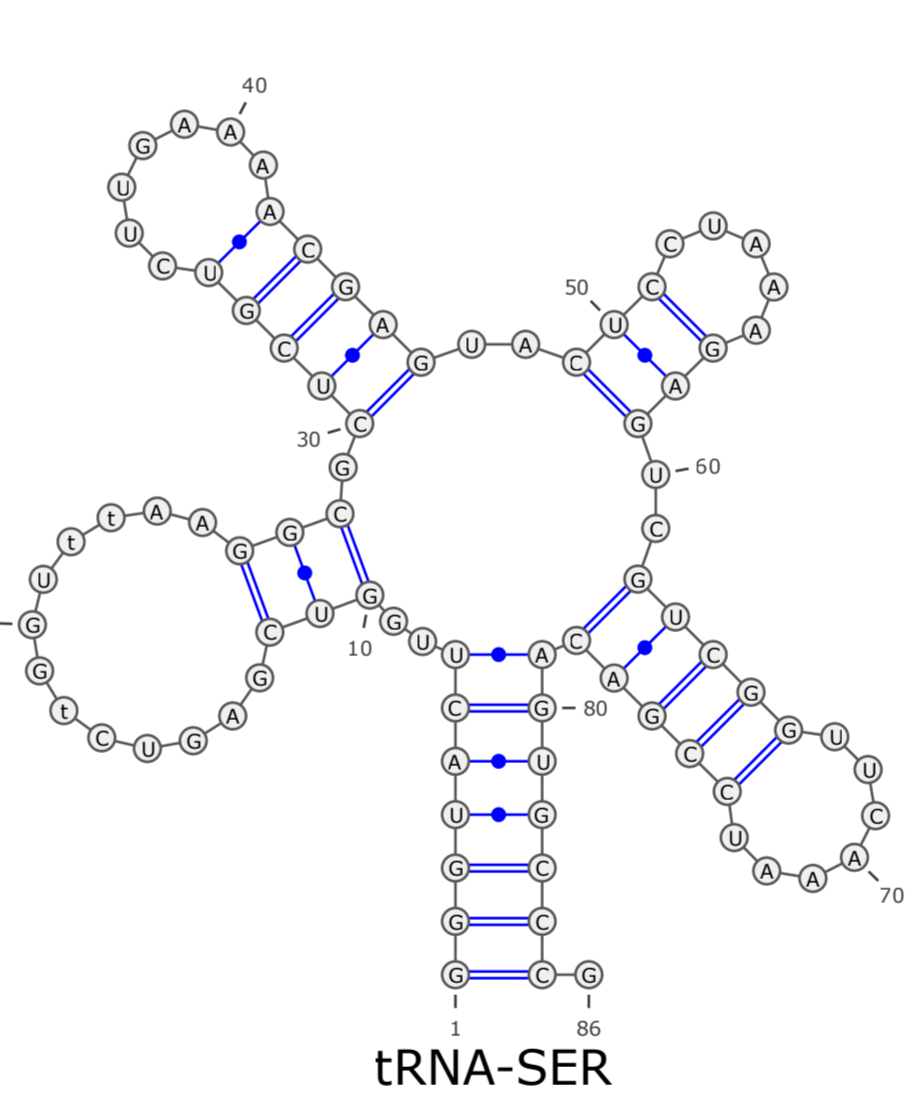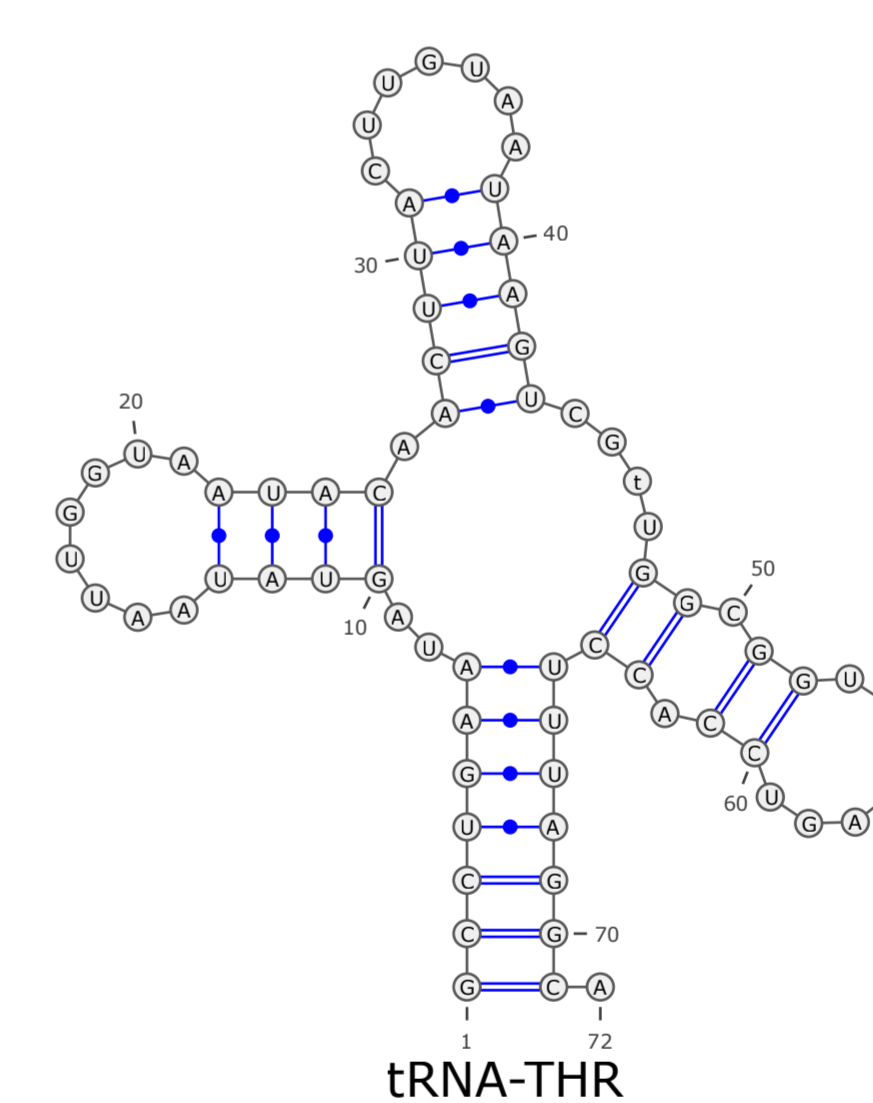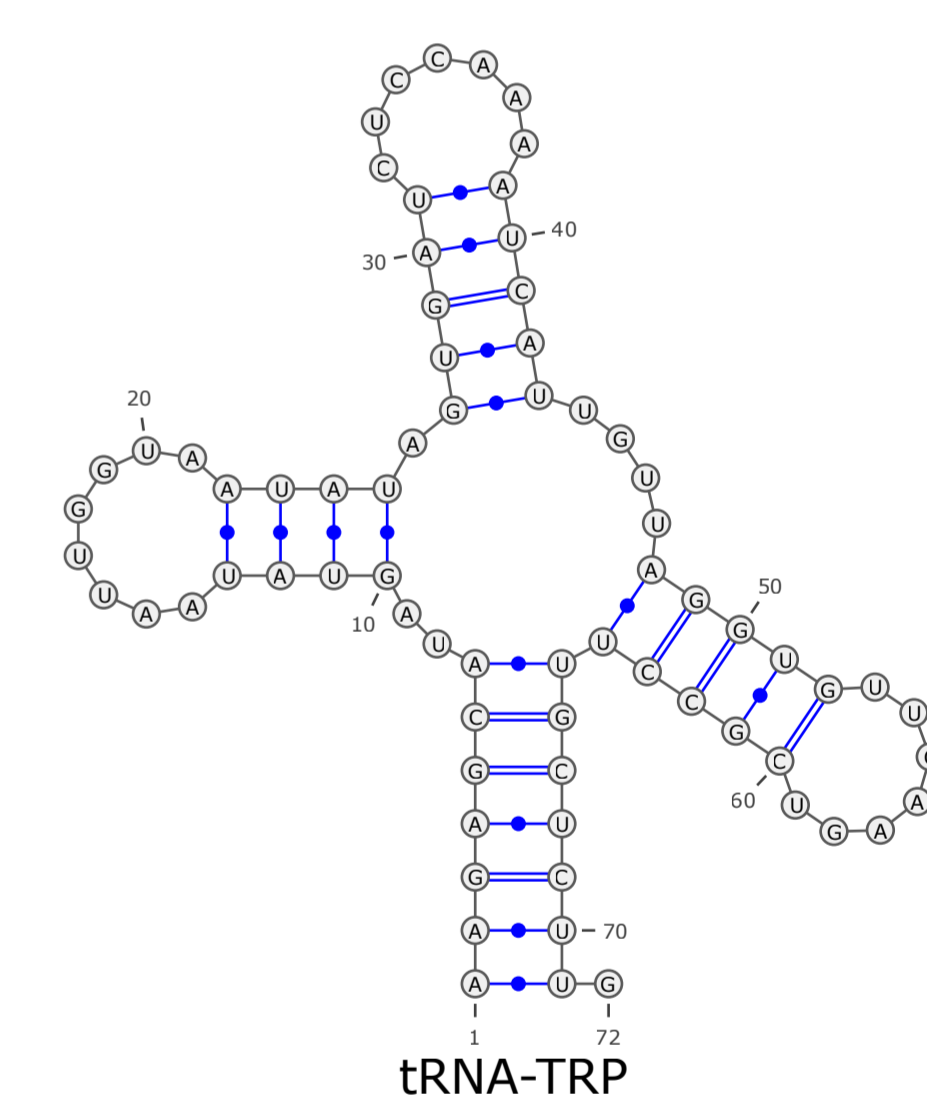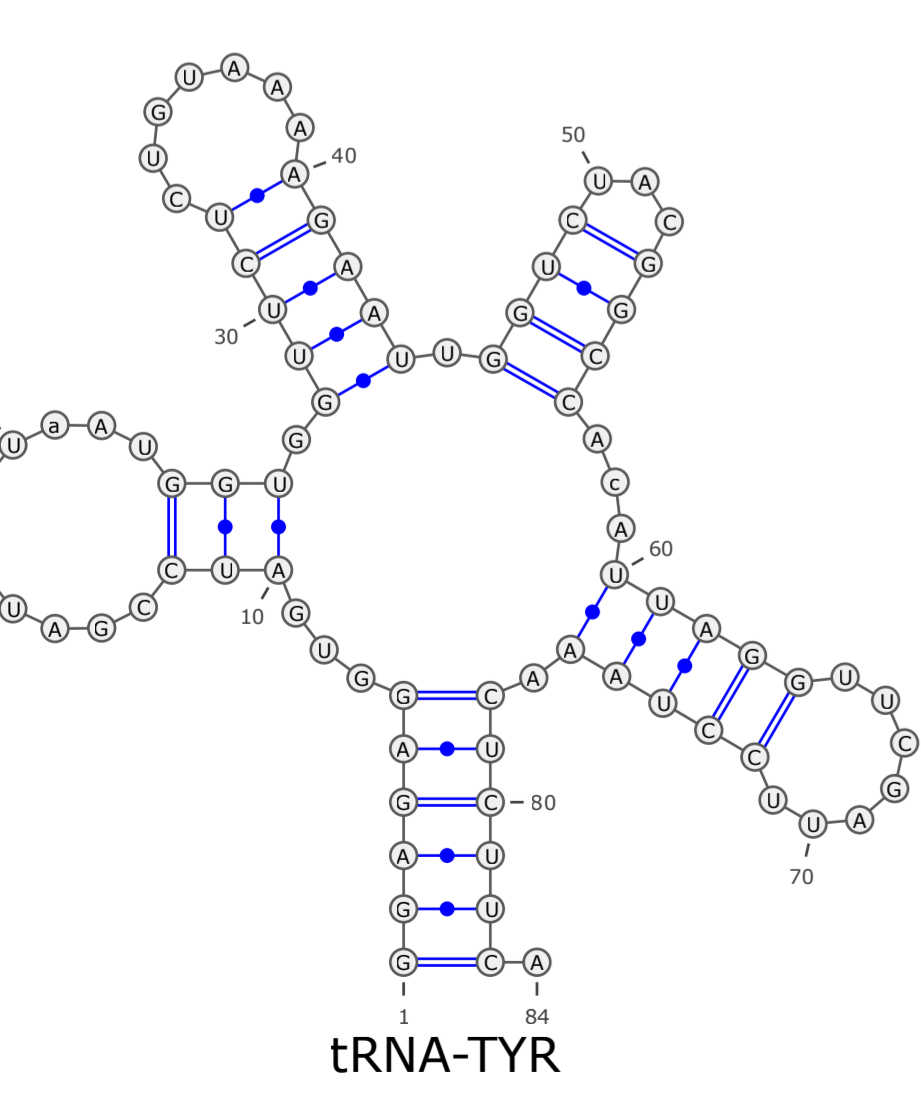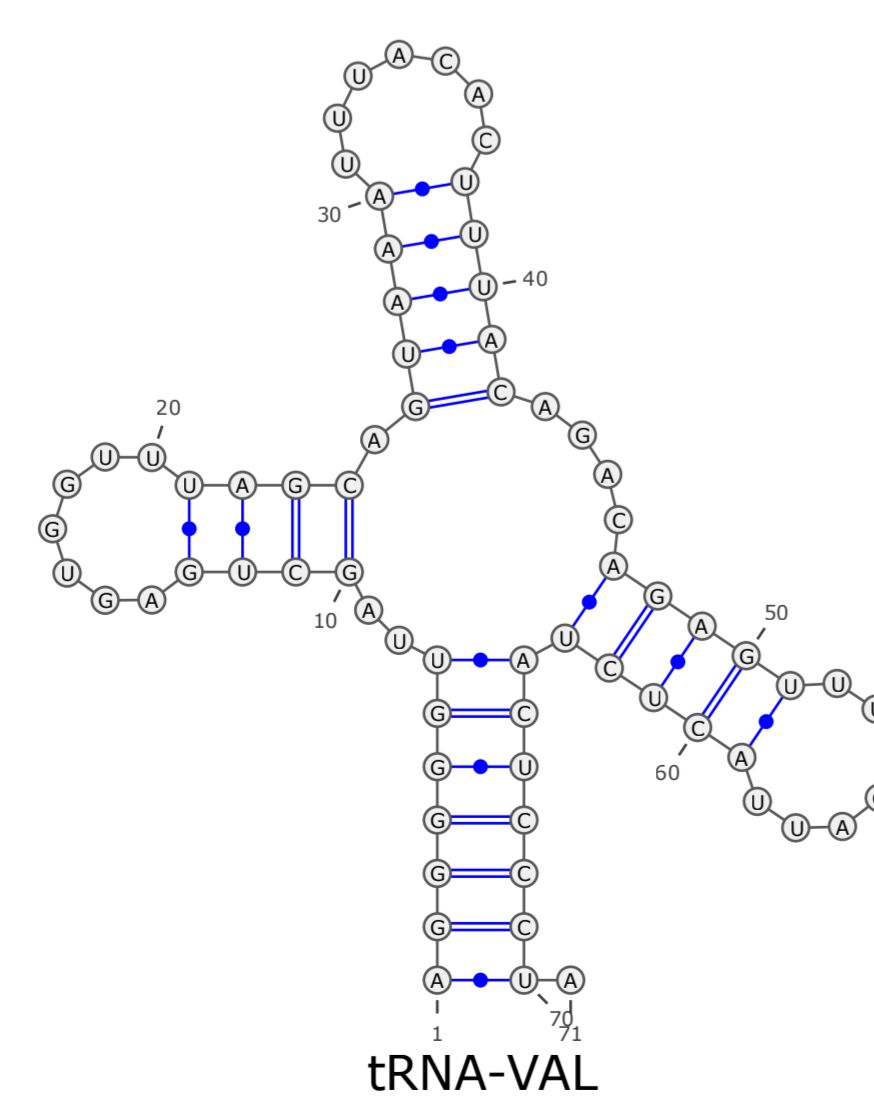

Supplement: Supplementary file 1 [file jof-11-00831-s001.zip › FIG6-Structure prediction/Figure S3-Chiua viridula.pdf]

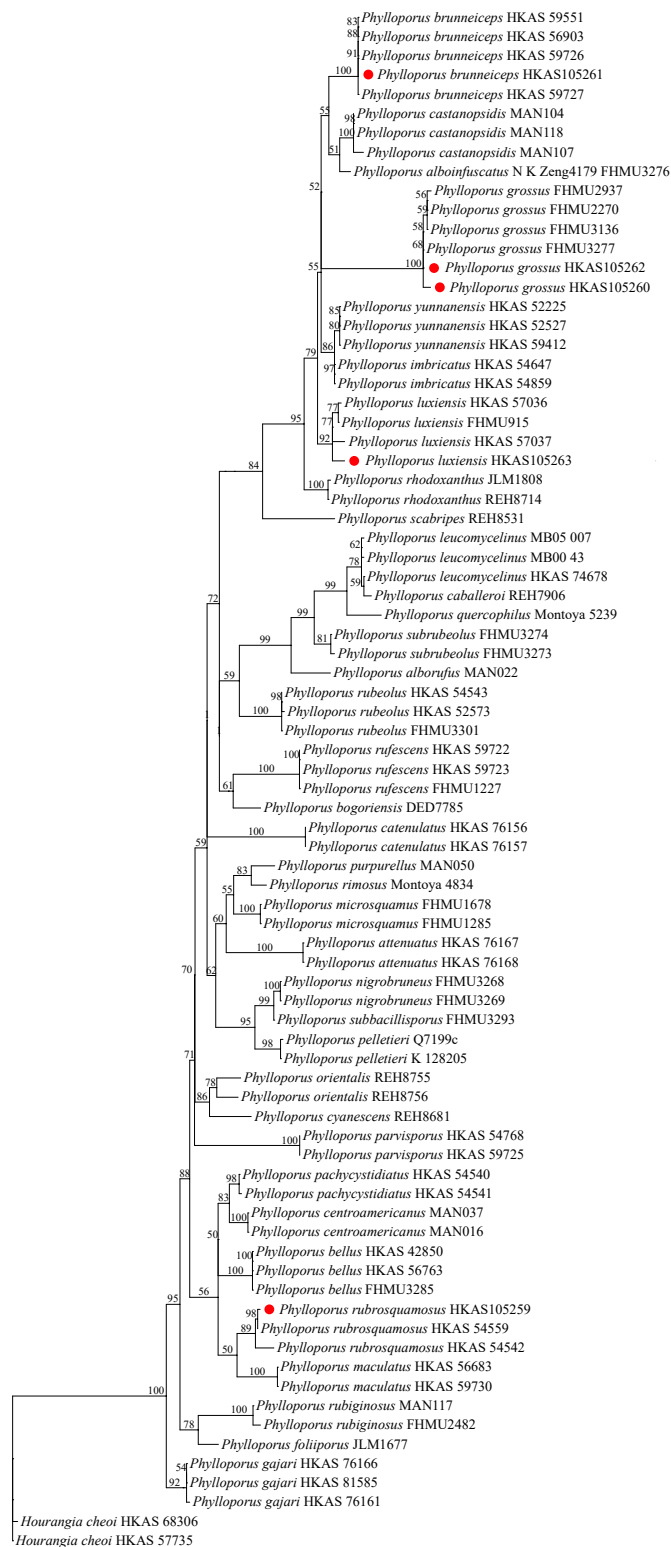

Tree scale: 0.1

Supplement: Supplementary file 1 [file jof-11-00831-s001.zip › FIG8-Phylogenetic relationships/Figure S9-Phylogenetic analysis of Phylloporus.pdf]
